# Inference of drug off-target effects on cellular signaling using interactome-based deep learning

**DOI:** 10.1016/j.isci.2024.109509

**Published:** 2024-03-14

**Authors:** Nikolaos Meimetis, Douglas A. Lauffenburger, Avlant Nilsson

**Affiliations:** 1 Department of Biological Engineering, Massachusetts Institute of Technology, Cambridge, MA 02139, USA; 2 Department of Cell and Molecular Biology, SciLifeLab, Karolinska Institutet, Stockholm, Sweden; 3 Department of Biology and Biological Engineering, Chalmers University of Technology, Gothenburg, SE 41296, Sweden

**Keywords:** Bioinformatics, Biological sciences, Health informatics, Health sciences, Medical informatics, Natural sciences, Pharmacology

## Abstract

Many diseases emerge from dysregulated cellular signaling, and drugs are often designed to target specific signaling proteins. Off-target effects are, however, common and may ultimately result in failed clinical trials. Here we develop a computer model of the cell’s transcriptional response to drugs for improved understanding of their mechanisms of action. The model is based on ensembles of artificial neural networks and simultaneously infers drug-target interactions and their downstream effects on intracellular signaling. With this, it predicts transcription factors’ activities, while recovering known drug-target interactions and inferring many new ones, which we validate with an independent dataset. As a case study, we analyze the effects of the drug Lestaurtinib on downstream signaling. Alongside its intended target, FLT3, the model predicts an inhibition of CDK2 that enhances the downregulation of the cell cycle-critical transcription factor FOXM1. Our approach can therefore enhance our understanding of drug signaling for therapeutic design.

## Highlights

• A deep learning model that predicts the transcriptional effects of drugs
• Automatically infers off-target effects of drugs
• Decouples off- and on-target effects on transcription
• Extracts causal signaling networks

## Graphical abstract

**Figure:**
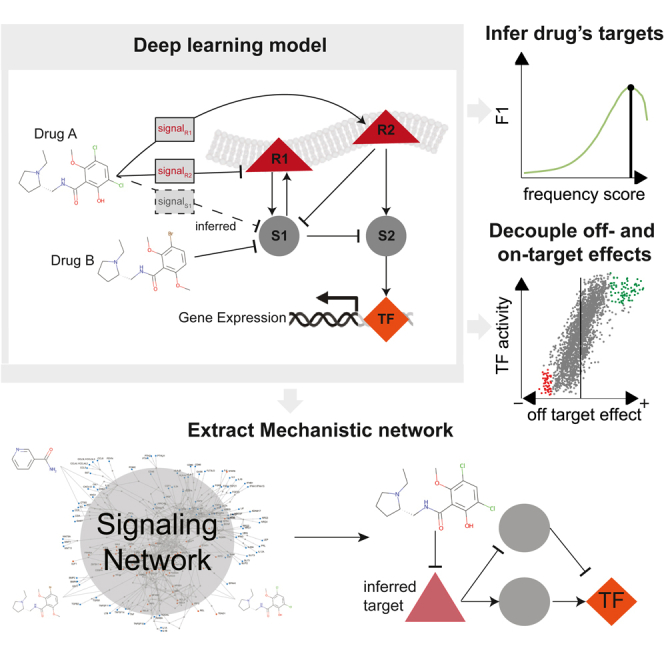

## Introduction

In many diseases, such as cancer, alterations in gene expression or protein function lead to dysregulated intracellular signaling, with pathological effects.^1^^,^^2^^,^^3^^,^^4^ This may be counteracted by perturbing cellular signaling using drugs, in particular small molecules that have been used for decades to revert cells to a healthy state or kill cancerous cells,^1^ e.g., inhibition of Ras-mediated signaling in anticancer therapy.^5^ This approach aims to affect signaling through specific drug-target interactions, but the drugs do not necessarily function through their proposed mechanism of action (MoA),^6^ and off-target effects are common.^7^ Understanding the contributions of on- and off-target effects of drugs is important for the development of safe therapeutics and their success in the clinic.

Systems pharmacology approaches have been developed to decipher the MoA of drugs. Several of these utilize data from chemical perturbation experiments.^8^^,^^9^^,^^10^ For example, these approaches may utilize the transcriptomic profiles of perturbed cells to identify key genes associated with specific therapeutic or adverse effects^11^ or elucidate their signaling mechanism based on their gene expression profile and large datasets of known drug-target interactions.^12^^,^^13^ With the advent of machine learning (ML) and large-scale high-throughput screening (HTS) datasets, such as the L1000 dataset,^14^ consisting of thousands of drug perturbations tested on cancer cell lines, these approaches have become more efficient, e.g., leading to the identification of novel potential therapeutic targets^15^ and to direct characterization of the transcriptomic profile of perturbations.^16^ However, these approaches do not explicitly model the signal propagation that underlies these effects and their predictions can therefore not be directly interpreted in terms of molecular mechanisms.

Signaling networks provide a scaffold to comprehensively describe a drug’s MoA. Molecular networks have been used to agglomerate signature MoA predictions^17^ as the basis for large-scale computer models to facilitate genome-scale simulations of perturbations.^18^^,^^19^ This has become feasible due to the extensive characterization of the intracellular signaling network^20^^,^^21^ and improvements in parameter fitting methods. For example, in early work, Saez-Rodriguez et al.^22^ used Boolean modeling on a small-scale signaling network to predict inflammatory signaling in HEPG2 cell lines while inferring interactions that were missing from the initial network. In more recent work, Fröhlich et al.^23^ developed a large-scale mechanistic model using ordinary differential equations (ODEs) to predict the response to drug perturbations in 120 different cell lines. Alongside the signal network, this model relied on a sparse network of drug-signaling protein interactions that was manually curated from the literature. However, despite major advances, the parameter fitting of ODE-based models could require problematically long computational times when applied to genome-scale networks.

Artificial neural networks (ANNs) allow for rapid parametrization of large-scale models. These are now being used for predictions in many areas of biology,^24^ e.g., for protein folding,^25^ histology,^26^ and response to therapy in cancer.^27^ A limitation of ANNs in their default formulation is that they are black-box models, which do not allow for direct interpretation of their predictions. This may be particularly problematic when predicting the effects of drugs since understanding their MoA is central to safety and establishing trust in a treatment. However, interpretable ANNs have been established that are constrained to only allow mechanistically plausible predictions, based on prior knowledge networks.^28^^,^^29^ These have been used to predict receptor stimulation from gene expression data^28^ and the effects of ligands on transcription factor (TF) activity.^29^ We recently established a modeling framework, termed, large-scale knowledge embedded artificial signaling network (LEMBAS), based on recurrent neural networks (RNNs), that simulates intracellular signal propagation including feedback loops.^29^ For this, we took advantage of both a prior knowledge network of signal transduction and a transcriptional regulatory network.^30^ The latter was used to infer TF activity from gene expression using the VIPER algorithm,^31^ which tests for regulon enrichment on gene expression signatures. We also adapted the LEMBAS framework to replicate the prediction of drug responses from the Fröhlich study^23^ with indistinguishable accuracy and much faster parameterization time. However, both of these approaches depend on prior knowledge of drug-target interactions and were not designed to infer new drug targets.

Because it is improbable that all drug-target interactions have already been discovered, in particular for newly developed drugs, inference of new interactions could be of importance to completely explain the effects of drugs. Many different ML approaches have been developed to infer new potential interactions using bioactivity data, dose responses, and large databases of prior knowledge containing known drug-target interactions.^15^^,^^32^^,^^33^^,^^34^ However, current ML approaches focus on inferring single drug-target interactions or binding affinities, based on either chemical structures^35^^,^^36^ or gene expression profiles,^15^ without fully utilizing the signaling network. They thus lack direct interpretability and the ability to comprehensively describe the signaling cascades arising from off-target MoA.

Here we have developed an approach to predict network-wide signaling responses to drugs that considers both on- and off-target effects. We expand the ANN-based signaling framework^29^ to combine a prior knowledge network of signaling,^20^ a network of known drug-target interactions, and the drugs' chemical structure similarity with other drugs, to simultaneously infer drug-target interactions and simulate the regulatory effect of known and inferred interactions in drug perturbation experiments. We use publicly available data on the transcriptomic response to drug perturbations that we process further to infer TF activities. We use the data to train cell-line-specific signaling models that we use to identify potential off-target effects of drugs alongside MoAs that can explain them. We validate the inferred interactions using an independent dataset and explore some of the predicted MoAs using *in silico* simulations and public gene knockout data.

## Results

### A model for predicting network-wide signaling of drugs via modeling of on- and off-target effects

**Figure 1:**
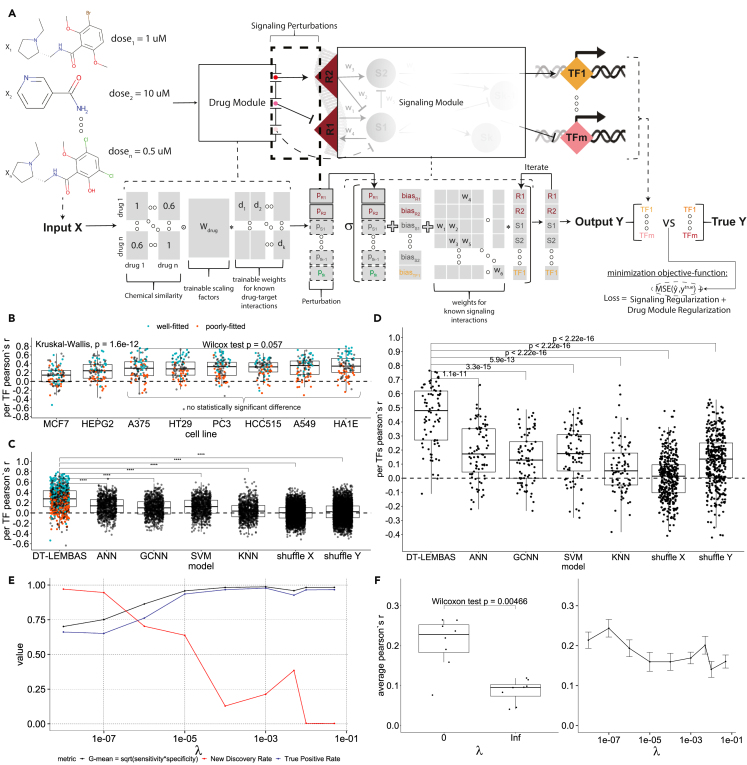
Model architecture and basic performance metrics (A) DT-LEMBAS’ model architecture, consisting of two interconnected sub-modules: 1) a drug module that generates drug signaling on nodes of the network, via drug-target interaction inference, based on known drug-target interactions and chemical similarity with other drugs and 2) a LEMBAS-based recurrent ANN, modeling intracellular signaling. The input concentration matrix of available drugs is first multiplied by the element-wise product between the pre-calculated chemical similarity and a trainable weight matrix ($W_{drug}$), acting as a trainable scaler of chemical similarity (see STAR methods for more details). (B) Performance of our approach in different cell lines. (C) Performance comparison in validation sets with standard machine learning approaches, for every TF in every cell line, using Pearson’s r between predicted and actual TF activity. The model is also compared to two randomized models, derived by shuffling the inputs (X) and outputs (Y) during training. The Pearson correlation distribution of DT-LEMBAS and that of every other model were compared using a two-sided unpaired Wilcoxon test; asterisks indicate significance level defined as: ∗∗∗∗p<=10^−4^, ∗∗∗p<=10^−3^, ∗∗p<=10^−2^, ∗p<=0.05, and ns for p > 0.05. (D) Performance comparison in validation sets, by comparing only the TFs that were well fitted (here top 10%) during training. (E) The optimal geometric mean of sensitivity and specificity for inferring drug-target interactions, and the NDR and TPR for the same gradient cutoff, at different levels of regularization. (F) Average performance across all TFs in every cell line for different levels of regularization. The error bars denote a deviation of one standard error (SE) from the mean. A two-sided unpaired Wilcoxon test was used to compare “*infinite”* and “*0”* regularizations. In all boxplots, the centerline denotes the median, the bounds of the box denote the 1st and 3rd quantiles, and the whiskers denote points not being further from the median than 1.5× interquartile range (IQR).

We developed an approach (denoted as DT-LEMBAS) for predicting the regulatory effect of drug perturbations, while simultaneously inferring unknown drug-target interactions (Figure 1A and details in STAR methods section). The model consists of two interconnected sub-modules. The first module takes drugs’ concentration as input, multiplies the concentrations matrix with the element-wise product between the pre-calculated chemical similarity and a trainable weight matrix ($W_{drug}$), which acts as a trainable scaler of chemical similarity, and generates their signaling effect on drug targets as output. The second module is LEMBAS, a published model of intracellular signaling that takes a drug’s signaling effects, generated by the drug module as input, and returns the TF activity as output.^29^ LEMBAS is a recurrent ANN model of intracellular signaling, where the connections are based on prior knowledge of the intracellular signaling network, thereby constraining the model to mechanistically plausible predictions.

In the case of drug perturbations, such as treatment with small molecules, the prior knowledge of the drugs' targets may be incomplete, thus creating the need to infer potential drug-target interactions and the off-target signal that can be induced on the targets. To achieve this we utilize both known drug-target interaction information, taken from the Broad Institute Repurposing Hub,^37^ and pre-calculated chemical similarity between drugs, using their ECFP4 molecular fingerprints^38^ to quantitatively calculate their pairwise Tanimoto similarity. We encode the drug-target interactions as a trainable spare weight matrix and the chemical similarity as a drug-drug similarity matrix, forming a pre-defined drug/target space (Figure 1A). The concentration of a drug of interest is taken as input, and, based on the similarity with other drugs and its known targets, the module is allowed to infer potential drug-target interactions, via the utilization of the trainable weights and prior knowledge of the drug module, leading to signaling effects that are propagated as input signal to the LEMBAS module. We train the combined model to fit TF activity data while minimizing a few regularization terms, aimed at controlling the number of new inferred drug-target interactions from the drug module, alongside regularizations and other priors previously developed for LEMBAS^29^ (see STAR methods). The hyper-parameters to build and train this framework can be found in Table S1.

### Performance in predicting activities of individual TFs

To train our model, and evaluate its performance in predicting activities of individual TFs, we used gene expression data from the L1000 dataset.^14^ As the purpose of this study is to examine the short-term signaling of drugs, to avoid self-regulatory effects we excluded long-period experiments and used only perturbations where cell lines were treated with a drug for less than 12 h.

As ANNs are known to overfit the data, before performing any subsequent downstream analysis of the predicted MoA of drug perturbations, it is useful to determine which TFs the model is able to predict correctly. Similarly, it is necessary to ensure that the predictions of the drug module generalize sufficiently well for the inferred drug-target interactions to be trusted. Cross-validation is a common validation strategy, where some of the data are withheld from the training set; however, in this dataset, drugs only appear once per cell line, meaning that there would be no training data available for the drug using this approach. An additional challenge for ML methods using chemical representations is misleading high performance due to memorization of similar structures.^39^

To circumvent these issues, we devised a validation strategy (Figure S2) to evaluate the performance of the model in predicting TFs’ activities, which makes use of data from different cell lines to construct the drug module, while applying a cross-validation schema to the signaling module. Specifically, we first filter our data to select cell lines with at least 400 drugs tested on them and then keep those that have altogether at least 200 drugs in common (more details in STAR methods and Figure S1, selection procedure), resulting in 9 cell lines. Then, from these 9 cell lines, we use the one (VCAP) with the most samples available (from the common drugs) to train a full model. For the remaining 8 cell lines we keep the drug module unchanged and train only the signaling part of the model using 80% of the drugs available while validating using the remaining 20%, i.e., 5-fold cross-validation. The hypothesis is that, if the drug module is not general enough and cannot generate a general enough signal on potential targets, then, for the 20% of test drugs, now that the signaling module is re-trained and changed, the model will have poor performance. Because ML models tend to memorize chemical structures,^39^ we confirm that test drugs are generally dissimilar in their chemical structure from training drugs, thereby avoiding information leakage from similar drugs (Figure S3). A Tanimoto similarity threshold of 0.5–0.6 is usually enough to consider two drugs similar when using ECFP4 fingerprints with this similarity threshold.^40^ We find that the model predicts the activities of many TFs with high accuracy, and a similar behavior is observed across all cell lines (Figure 1B). Notably, there is a big variation in the performance between individual TFs, and, since we are aiming to utilize some of them for downstream analysis, it would be useful to find a principled way to identify high-performing TFs. We hypothesize that some TFs will be poorly fitted by the model during training due to various reasons, e.g., because their data may be noisy, because they may not be contributing to the transcriptomic profile of the cell, or because their activity cannot be explained by the prior knowledge signaling network. Indeed, TFs that were well fitted during training (rank ≤25% in training based on Pearson correlation) were overrepresented among the high-performing TFs in validation, while most TFs that were poorly fitted (rank ≥75% in training) also performed poorly in validation (Figures 1C and S7). Similar results can be observed if the drug module is initially trained in other cell lines such as A375 and A549 (Figure S6).

To determine if these validation results were in line with what could be expected given the data, we benchmarked them against four basic ML techniques. Specifically, we compared the models’ ability to predict TF activities with: 1) an ensemble of 50 simple feedforward ANNs that take as input the ECFP4 molecular fingerprints of drugs, 2) an ensemble of 50 graph convolutional neural networks (GCNNs)^41^ representing drugs’ chemical structures as graphs, 3) an ensemble of 50 support vector machines (SVMs), and 4) an ensemble of 50 k-nearest neighbors (KNN) models. Additionally, as two types of null models, we trained two models where we 1) shuffled the input matrix of drug concentrations (X) during the training of the drug module, thereby generating a randomized drug module, or 2) we shuffled the outputs (Y) during re-training of the signaling part for each cell line, generating a randomly weighted signaling network. Our model outperformed these approaches, as well as the null models, based on a non-parametric two-sample Wilcoxon test (Figure 1C). The validation performance for the top 10% fitted TFs during training was generally high (Figure 1D), achieving an average Pearson correlation of ∼0.5 (Figure S4B), with the performance of some TFs higher than ∼0.8 and p values ≤ $10^{-6}$ (see Figure S5 for the adjusted p values for all of the correlations). This suggests that we can rely on the predictions for some of the TFs in our subsequent analysis.

### Constraining the number of inferred interactions via weight regularization

We make use of the assumption that drugs will not interact with most targets to make more specific predictions. To control the number of inferred interactions, we utilized an L2-based regularization scheme for the weights of the drug module such that infinite regularization constrains the module to only make use of known drug-target interactions and zero regularization allows every possible interaction without penalty (see STAR methods). Since we cannot know in advance which targets a drug does not affect (true negatives), we instead aim to find a good trade-off between sensitivity and specificity in inferring interactions, as well as prediction performance. We utilized an integrated gradient score approach^42^ (see STAR methods) to quantify the confidence in a drug affecting a target node in the signaling network, and we inferred interactions by identifying a cutoff for the absolute value of that score (see STAR methods). With increasing regularization, the trade-off between sensitivity and specificity saturates (Figure 1E) when inspecting their geometric mean (optimal G-mean) at the cutoff that maximizes it. To quantify the amount of interactions at different regularization levels we define a metric, new discovery rate (NDR), as the number of new interactions inferred divided by the number of total interactions inferred by the model. We find that, for increasing regularization levels, this metric decreases and slowly goes to zero, as intended (Figure 1E). Meanwhile, for increasing regularization levels the true positive rate (TPR) increases and saturates, indicating that with increasing regularization the model depends more on prior knowledge, and as intended it does not exclude a lot of prior knowledge interactions to reduce the total inferred interactions (Figure 1E). Similarly, for every regularization level at different gradient score thresholds, the G-mean and TPR increase and start saturating after λ = 1E-04 while the NDR decreases until it becomes almost zero (Figure S8A). This result appears to be robust to using a different error-based method to infer interactions (see STAR methods, and Figure S8B). Finally, for the average performance of individual models (not the ensemble) trained using different regularization levels, we observe that zero regularization outperforms infinite regularization (Figure 1F). This indicates that the addition of inferred interactions contributes to the model’s predictive power. However, there is not a clear trend for intermediate levels of regularization; nevertheless, it seems that, for the regularization level λ = 5E-03, the performance is slightly higher than its neighboring levels (Figure 1F). This could perhaps be due to the locally higher NDR at that regularization level (Figures 1E and S7). Because of this, alongside the higher performance, we selected this regularization level for the models trained in this study (including the models in Figure 1C).

### Inferring drug-target interactions with integrated gradient scores

The model is constructed to allow inference interactions that are not part of the prior knowledge to better explain transcriptional data. To extract which drug-target interactions have been inferred, we use integrated gradients to assign an importance score to each interaction (see STAR methods). In the case of a linear drug module, used in this study, the score is proportional to the module’s weights (Figure S9). A negative score corresponds to a potential inhibition of a target node from a drug of interest, while a positive score corresponds to activation.

**Figure 2:**
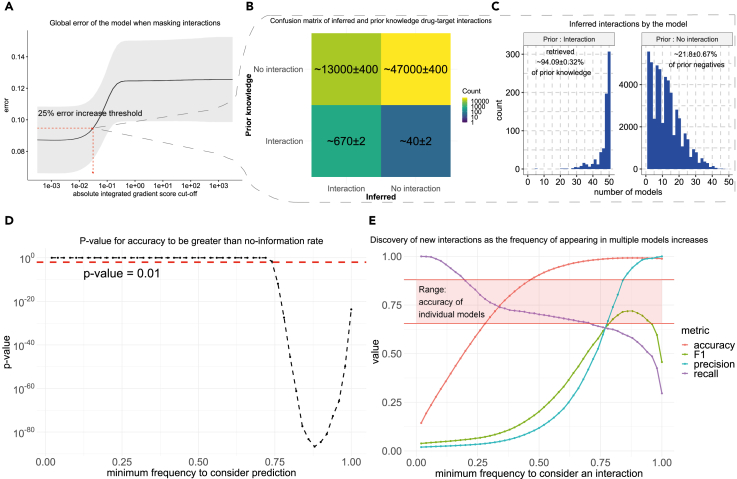
Inferring drug-target interactions in the A375 cell line from the drug module (A) Error of the model as more important drug-target interactions, according to their integrated gradient score, are removed. The shaded area denotes a deviation of one standard error (SE) from the mean. (B) Average confusion matrix from 50 trained models for the inferred drug-target interactions. (C) Percentage of prior knowledge drug-target interactions and previously unknown interactions retrieved, and their corresponding frequency of appearance in multiple models. (D) p values from comparing accuracy with the accuracy obtained by assigning everything to the predominant class (no information rate, NIR), for multiple frequency scores, in this imbalanced dataset where most drugs do not interact with most targets. (E) Classification performance of our approach by considering as ground truth the interactions contained both in the Broad Institute Repurposing Hub^37^ and in DrugBank.^13^ Performance is calculated for an increasing frequency of an interaction appearing in multiple models.

To identify a cutoff level for the score we investigated how the model’s performance decreases as more interactions are removed (see STAR methods). Briefly, for each drug, we successively removed more interactions, based on the absolute value of the score, and determined how this affects the error of the model in predicting the activity of all TFs (Figure 2A). As can be expected, removing interactions with low scores did not affect the error of the model, while, at some critical level, the error sharply increased and finally plateaued. This means that the model’s low-scoring interactions are not needed to explain the TF activity, while, for high-scoring interactions, the error of the trained model increases dramatically. For each drug in each trained model, we define the cutoff at a 25% percentage increase in error. This approach was chosen because of its high NDR (Figure S8) and because it allowed us to infer many new interactions which at the same time are necessary for the model to correctly predict the TF activity. Subsequently, we utilized the ensemble of models to also score the confidence in inferring an interaction by using the frequency of appearance in multiple models.

The first step to evaluating the validity of this approach to infer drug-target interactions is whether it can retrieve most of the prior knowledge interactions (on-target effects), as these are expected to be able to explain at a large level the observed transcriptional profile. Indeed, when training a model for the A375 cell line, we can retrieve most of the interactions in the prior knowledge used in training the model while also inferring approximately 13,000 more interactions (Figures 2B and 2C), which can potentially be undiscovered direct drug-target interactions, indirect effects, or false interactions (false positives). It seems that prior knowledge interactions are inferred by most of the models in the ensembles, while undiscovered interactions appear mostly with low frequency, with some of them appearing in many models (Figure 2C). We observe similar results when training models and inferring drug-target interactions using the A549 and VCAP cell lines (Figure S10). Based on this, we hypothesized that it could be possible to predict if an inferred interaction is a true direct interaction, based on the number of times it was inferred by different models.

### Evaluating the inference of direct interactions by an ensemble of models

We make use of an independent drug-target interaction database to evaluate the predictive power of the model. While we know the existing true drug-target interactions (true positives), the true negatives are unknown, and it is not clear to which extent predicted interactions can be trusted. To partially overcome this limitation, we make use of a more comprehensive database, DrugBank,^13^ for the drugs present in our trained framework, to establish a set of true interactions that were not present in the prior knowledge used to construct the model. We then attempt to predict these interactions depending on how frequently they are inferred in our models. From a frequency of 0.75 (interaction inferred for 37 out of 50 models), there is a statistically significant difference between the model’s accuracy in predicting drug-target interactions and the null accuracy obtained by assigning everything to the predominant class (no information rate, NIR) (Figure 2D). In addition to accuracy, we also consider the following evaluation metrics: precision, recall, and the F1 score, which is the trade-off of precision and recall for imbalanced data (Figure 2E).

We find that including interactions that appear in any model is too lenient, resulting in poor precision (1.97%) and accuracy (14.35%), and the predictions of individual models do not perform better than chance with an accuracy of ∼75%. However, for increasingly frequent interactions both precision and accuracy increase markedly, with perfect precision (100%) for the most frequent predictions (Figure 2E). At high inference frequencies, recall decreases drastically, meaning that the inference threshold may be too strict. The F1 score (which is a trade-off between precision and recall, given by the formula $F1=2*\frac{\text{precision}*\text{recall}}{\text{precision}+\text{recall}}$) generally increases until it reaches a maximum of 71.84% for an interaction appearing with a frequency score of 0.88 (44 out of 50 models), and then it starts decreasing as precision saturates, given there are only a few known drug-target interactions, while very high thresholds for inferring interactions are too strict and recall continues decreasing. The aforementioned frequency score is the same number of models which corresponds to the higher accuracy (99.22%) and the lowest p value signifying statistical significance in the difference between accuracy (99.22%) and NIR. We observe similar results for models trained on A549 and VCAP cell lines (Figure S11). This evaluation showed a higher accuracy than we could expect from naively guessing that an interaction does not exist, which supports the hypothesis that interactions appearing in multiple models are more likely to correspond to direct interactions which enables the potential for inferring novel drug-target interactions. We provide all inferred drug-target interactions alongside frequency scores in Data File S1; e.g., Dacinostat, a known histone deacetylase inhibitor, is found in both A375 and A549 and VCAP cell lines, by more than 40 models, to interact with KDR which is a type III receptor tyrosine kinase. Generally, the frequency of appearance of drug-targets interactions is correlated across the three cell lines (Figure S12), while there seems to be a strong consensus between cell lines for high-frequency interactions, although there are many low-frequency interactions inferred in all cell line models (Figure S12).

We tested if the inferred off-target effects could help explain the lethality of drugs. Off-target effects are primarily thought to cause side effects, but, instead, they may contribute to the drug’s efficacy in some cases.^8^^,^^43^ We tested if the inferred targets could help predict the lethality of drugs tested on the 9 different cell lines in our study that were also present in the NCI60 drug screen.^44^ The NCI60 cell line panel was developed as an anticancer drug efficacy screen and consists of the molecular profiles of the 60 core human cell lines as well as the dose-response outcomes from applying thousands of drugs. The dataset provides similar EC50 values (half-maximal effective concentrations) as other drug screen datasets and cannot be considered an outlier dataset (Figure S13). Inspired by Vijay and Gujral who developed an ANN model to predict changes in cell migration of cancer cells using drugs’ target profile,^45^ we conducted an analysis where we trained 10 different models (LASSO, ridge regression, elastic net, random forest, XGBoost Tree, neural network, regression SVM with a linear kernel, Gaussian process, KNN, and a linear regression model) to predict lethality using the drug targets and cell line identity as input. We trained these 10 models using both the prior knowledge of drug-target interactions and the optimal threshold for inferring interactions as identified in Figures 2D, S11C, and S11D, as well as multiple thresholds ranging from appearance in one model to appearance in all models. We utilized a leave-one-out-cross-validation (LOOCV) procedure, where during training a drug was removed (if its targets appeared at least once in some other drug in the training) across all the cell lines where it was tested. We observe that, generally, all models outperform randomized models trained on data with shuffled labels (Figure S14). Whether a model performs better when using the prior knowledge or not is specific to that model (Figures S14C and S14E), but generally across all models there is not a significant difference between using the prior knowledge and the selected threshold for inferring interactions (as identified by Figures 2D, S11C, and S11D), while there exists some threshold which leads to a statistically significant improvement in predicting drug lethality (Figure S15). In general, though, it seems for the data used here, the on-target effects can already fully explain the lethality observed, and that the inferred interactions do not contribute further to the performance (Figure S15). Interestingly, when using the best-performing model which also consistently appears in the top five models across the different input types (LASSO), despite the lack of difference in performance, MAPK12 is selected by LASSO in every LOOCV split. MAPK12, which is a target not affected by any of the 15 drugs present in this lethality case study, is a kinase, part of the mitogen-activated protein (MAP) kinase signal transduction pathway, which has been proposed as a potential therapeutic target.^46^^,^^47^ This could be an example of a potential off-target effect that could explain lethality.

### Identification of TFs regulated by off-target effects

**Figure 3:**
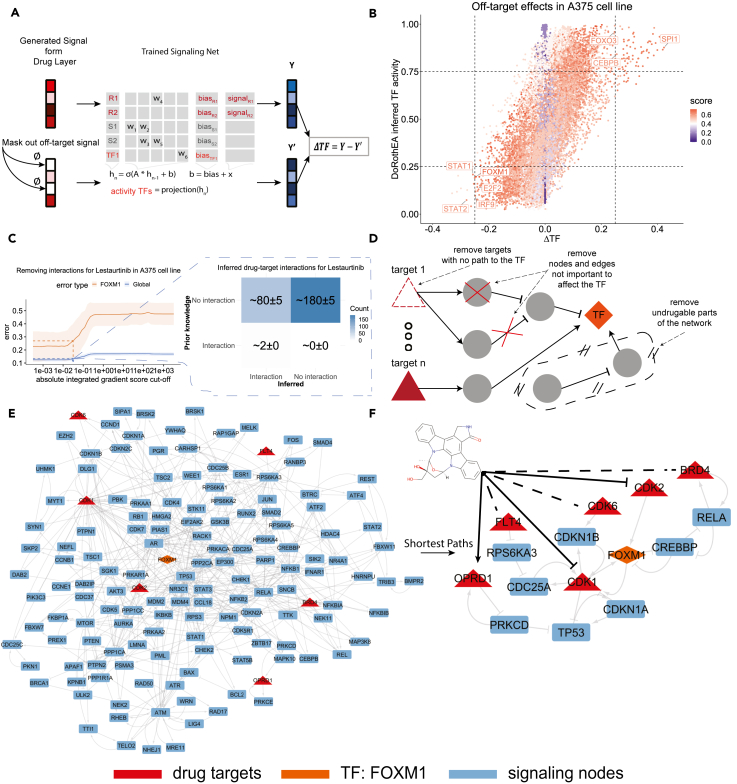
Process for interpreting off-target effects in A375 cell line, with a case study for Lestaurtinib’s effects on FOXM1 (A) The difference between the model’s predicted TF activity and the activity if the off-target signal is removed is used as a measure for the off-target effects on specific TFs. (B) The activity of the TFs compared with the predicted off-target effects alongside a confidence score from the average performance in training and validation. Each point corresponds to a specific drug-TF pair. (C) Inferring the drug-target interactions using multiple models and the global error approach previously discussed. Here the example of Lestaurtinib is shown. The shaded areas denote a deviation of one standard error (SE) from the mean. (D) The whole signaling network is trimmed by removing unimportant edges and nodes to control the TF of interest, stopping the process when there is no path from the inferred targets to the TF of interest. This process is repeated for every trained model and only frequently appearing edges and nodes are kept. (E) The trimmed ensemble network explaining the off-target effect that leads Lestaurtinib to inhibit more FOXM1, via previously unknown drug-target interactions. (F) A version of the trimmed network that only considers the simplest paths that connect every target to FOXM1. Lestaurtinib, according to the model, inhibits CDK1 and/or CDK2 kinases, while it interacts with an uncertain sign with CDK6, FLT4, and BRD4.

After establishing frequency thresholds for trusting predicted drug-target interactions, we make use of the signaling module to investigate their predicted MoA, in terms of inducing TF activity. We first identify whether the model predicts that there are marked off-target effects in response to a perturbation, by removing all of the input signal outputted by the drug module except the signal corresponding to the known targets and using it as input to predict the induced TF activities (Figure 3A). We consider the difference between the models’ original predicted TF activities and the ones where off-targets are masked out (ΔTF) as a proxy for the magnitude of the off-target effects on specific TFs. Samples where a TF has been activated (here considering activity ≥0.75) or inhibited (activity ≤0.25), and with a high off-target effect, are of interest for investigation (Figure 3B). When this contributes to the observed direction of TF regulation, it may be considered a perturbation with off-target effects. We further restrict our analysis to TFs whose activity is predicted well by the model (of the A375 cell line), by making sure that the average of the performance in validation and training of the model for that TF is higher than 0.6. An example of this is the case of the drug Lestaurtinib, where the model predicts an inhibitory off-target effect on FOXM1 (Figure 3B). FOXM1 is a TF critically associated with the cell cycle, considered a master regulator overexpressed in most human cancers.^48^ For this reason, we select FOXM1 and Lestaurtinib for further analysis, but more drugs that have an off-target effect on some TFs, in A375, A549, and VCAP cell lines, are provided in Data File S2, together with their activity, off-target, and performance score. In this file, we include all samples regardless of the magnitude of the off-target effect, together with the corresponding performance of each TF, apart from the activity and the off-target effect.

### A subnetwork explaining off-target effects of Lestaurtinib

We infer all off-target interactions for the drug Lestaurtinib. As previously described (Figure 2A), we infer drug-target interactions by progressively masking potential interactions based on their integrated gradient score and calculating the error of the models for predicting the activity of TFs, until a sharp increase in the error appears. We repeat this process just for Lestaurtinib inspecting the effects on each TF independently. Strikingly the error of FOXM1 follows a trend similar to the average error across all TFs (Figure 3C). Using this approach, we identify a cutoff for the gradient score and infer on average 82 potential interactions out of the 259 available in our training space per model (Figure 3C). Two targets of Lestaurtinib exist in the prior knowledge used to train the model, which are NTRK1 and FLT3, and both of them are retrieved for all of the models (50 of 50). Additionally, while not in the prior knowledge used for training, DrugBank has another two targets for Lestaurtinib in our target space: EGFR and ADRB1. These are inferred in 16 and 27 of the 50 models, respectively. This means that the model retrieves the prior knowledge most of the time and additionally infers many other interactions not in the training set that act to explain off-target effects.

We extract a subnetwork explaining the MoA effects of Lestaurtinib. After inferring new targets and identifying a TF with prominent off-target effects, we use the model to construct a smaller signaling network explaining the MoA for the off-target effects. For this we remove nodes and edges in the trained signaling network models that are not important for regulating the activity of the TF (Figure 3D). This is based on an importance score (see STAR methods) where nodes are iteratively removed until the removal of a node breaks the connection to the inferred targets. We use an ensemble approach where the final subnetwork is constructed by margining networks derived from each trained model, keeping only nodes and edges appearing in multiple models (see STAR methods).

We apply this process for the case of the effects of Lestaurtinib on FOXM1, resulting in a subnetwork of the intracellular signaling network that explains this off-target effect (Figure 3E). Although strongly reduced, this network is still relatively large and difficult to interpret. This may be due to multiple plausible mechanisms being explored simultaneously as a response to limited data together with L2 regularization limitations. Alternatively, this may indicate that it is necessary to include many interactions to fully explain the off-target effect that Lestaurtinib has on FOXM1 activity, and further reduction of the network would be an oversimplification. Applying the simplest path algorithm to the network from each inferred target (in red) toward FOXM1 (in orange), we find that inhibition of CDK1 and CDK2 could lead to the direct inhibition of FOXM1 (Figure 3F). According to the model (Figure 3E), Lestaurtinib can potentially inhibit FOXM1 by inhibiting CDK1 or/and CDK2, activating OPRD1 (with low certainty), and interacting in some uncertain manner with CDK6, BRD4, or FLT4, meaning that the smaller subnetwork contains feasible intracellular interactions that can indeed explain the off-target effect. Indeed it has been observed that FOXM1 can be activated by both CDK1^49^ and CDK2,^49^^,^^50^ meaning their inhibition could lead to inhibition of FOXM1, as proposed by the model. Additionally, while the interaction between Lestaurtinib and CDK2 is present in neither the prior knowledge used for training nor DrugBank, it has been seen in a comprehensive kinase inhibition study that Lestaurtinib indeed inhibits CDK2 with a K_d_ = 20 nM,^51^ which is markedly lower than the dose used in the L1000 study (10 μm). We note that CDK2 was identified as an interaction in 36 out of 50 models, bordering the previously identified threshold (of 37) for identifying true direct interactions with high performance (Figures 2E–2D). CDK1 is found in 35 out of 50 models while CDK6, BRD4, FLT4, and OPRD1 were found in only approximately half of the models. Taken together, this indicates that the model can be used to propose an MoA to explain the off-target effect that is biologically feasible and potentially true, which is also cell line specific, which may serve as a basis for designing therapeutic interventions or drug combinations to cancel or enhance this off-target effect.

### A case study of FOXM1 regulation by CDK2

**Figure 4:**
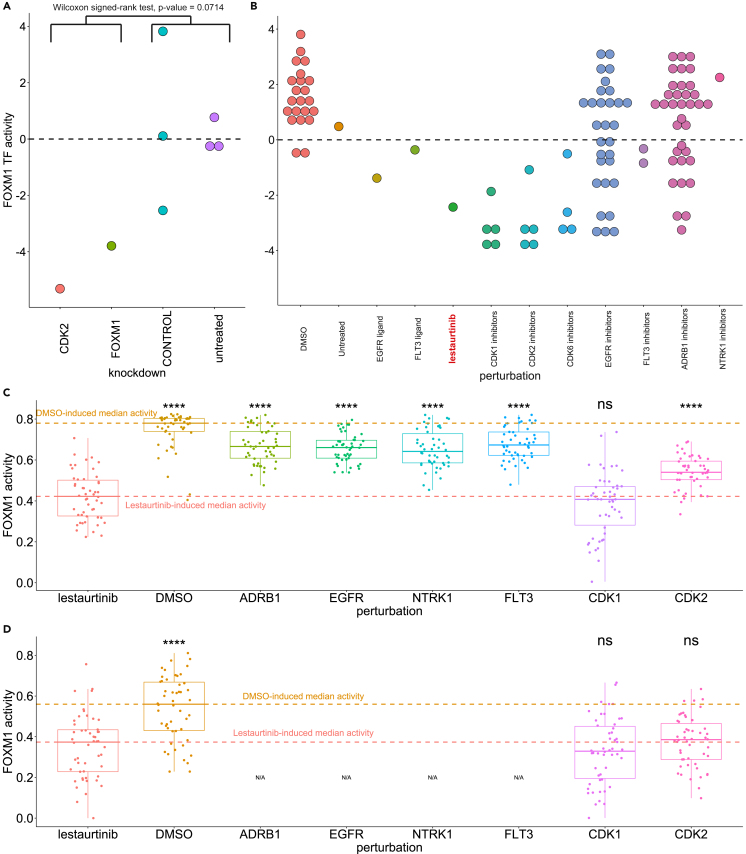
Validation of the predicted effects of Lestaurtinib on FOXM1 activity in the A375 cell line (A) Inferred activity after treatment with siRNA knockdowns of CDK2 and FOXM1 using public microarray data. (B) Inferred activity after treatment with ligands and inhibitors of Lestaurtinib targets, and CDKs in the L1000 dataset. (C) Predicted activity after an *in silico* knockdown. (D) Predicted FOXM1 activity using the inferred subnetwork explaining the MoA of the off-target effect of Lestaurtinib. Statistical comparisons in C and D were performed relative to Lestaurtinib, with a two-sided unpaired Wilcoxon test; asterisks indicate significance level defined as: ∗∗∗∗p ≤10-4, ∗∗∗p ≤10-3, ∗∗p ≤10-2, ∗p<=0.05, and ns for p > 0.05. In all boxplots, the centerline denotes the median, the bounds of the box denote the 1st and 3rd quantiles, and the whiskers denote points not being further from the median than 1.5× interquartile range (IQR).

Since the activation of FOXM1 by CDK2 and CDK1 has been experimentally demonstrated, it may serve as a useful case study for determining how well the different components in our approach recapitulate this effect. First, we inspect the inference of TF activity from gene expression data, using the DoRothEA regulon^30^ together with the VIPER algorithm.^31^ For this purpose, we retrieved Affymetrix microarray data, from the Gene Expression Omnibus (GEO),^52^ generated from A375 cells treated with small interfering RNAs (siRNAs) against various TFs and signaling molecules.^53^ We then inferred the activity of FOXM1 for the measured gene expression data for CDK2 and FOXM1 knockdown as well as untreated cells and control (inactive fluorescently labeled siRNAs) samples. We find that the inferred activity (*Z* scored) of FOXM1 when knocking down CDK2 is similar to a FOXM1 knockdown, while FOXM1 is way more inactive than in untreated cells (centered to zero as expected) in both cases (Figure 4A). Even though this published study has limited statistical power, it does indicate that our inferred activities in the L1000 recapitulate the relationship between CDK2 and FOXM1, thus corroborating the proposed off-target effect.

Secondly, we investigate whether the inhibition of the activity of FOXM1 by Lestaurtinib is indeed primarily achieved through inhibition of CDK2 and/or CDK1. For this purpose, we utilized data from ligand perturbations contained in the L1000 dataset but not used for training the model. From this, we inferred FOXM1 activity for ligand stimulation of the known targets of Lestaurtinib, EGFR and FLT3, and additionally for stimulation with other drugs that inhibit these targets as well as the two additional targets in DrugBank (ADRB1, NTRK1), and known inhibitors of CDK1, CDK2, and CDK6 (such as Alvocidib, AT-7519, and Kenpaullone). We made sure to select inhibitors with at most 10 targets to minimize the risk of regulation of FOXM1 through other targets. As expected, we find that the activity of FOXM1 in A375 when using Lestaurtinib is much more inhibited compared to DMSO-treated or untreated A375 cells. We also find that it is on a similar level as for known CDK1 and/or CDK2 inhibitors (four inhibit both CDK1 and CDK2 [Figure 4B]). Meanwhile, only a few of the ADRB1 inhibitors (another Lestaurtinib target) show a similar trend, and the rest of the known targets do not inhibit FOXM1 activity at a comparable level (or not at all). This further supports the proposed MoA of Lestaurtinib inhibiting FOXM1 through the off-target effect on CDK2.

### *In silico* knockdowns using the model

Finally, we investigate if *in silico* knockdown experiments by the model, using the proposed MoA network, recapitulate the similar effects on FOXM1 activity by Lestaurtinib, and CDK2 and CDK1 knockdowns. We induce a level of knockdown for a signaling node by assigning a large negative value as input. This way we knock down the known targets of Lestaurtinib (EGFR, NTRK1, ADRB1, FLT3) and CDK2 and CDK1, for all of the 50 trained signaling networks. Additionally, we model the signal generated by Lestaurtinib, signals masked to include either its on-target effects or off-target effects, as well as the signal from DMSO as control. We find that the activity of FOXM1 under Lestaurtinib is indeed much lower than DMSO, and that seems to be mostly due to the off-target effects (Figure 4C). We note that the on-target signal induced similar activity levels as knockdowns of any of the known targets of Lestaurtinib, indicating that the model can successfully recapitulate the on-target effects of Lestaurtinib on FOXM1, which, while lower than for DMSO, do not seem to strongly inhibit FOXM1 activity (Figure 4C). Furthermore, increasing the knockdown level for these nodes does not seem to induce much stronger inhibition of FOXM1 (Figures S16C and S16D), while knocking down CDK1 and CDK2 induces strong inhibition of FOXM1 and, depending on the knockdown strength, (Figures S16C and S16D) induces similar inhibition as Lestaurtinib (Figure 4C) or almost completely deactivates FOXM1 (Figures S16A and S16B). Notably, if we restrict the model to only the reduced subnetwork when conducting this *in silico* experiment, we observe a similar trend for the CDK1 and CDK2 knockdowns, suggesting that indeed the subnetwork is sufficient to explain the MoA of this off-target effect (Figure 4D). Taken together this case study serves as a proof of concept for the utilization of the models to generate *in silico* experiments to potentially identify therapeutic interventions to cancel the off-target effect.

### The performance and inferred off-target effects depend on the class of drug

Encouraged by the results of the Lestaurtinib case study, we analyzed two other FLT3-inhibiting drugs with our MoA inference procedure. Interestingly, Dovitinib, a FLT3 and growth factor receptor inhibitor, was also found to inhibit FOXM1 through off-target effects and to yield a similar MoA including the off-target inhibition of FOXM1 through inhibition of CDK1 and CDK2 (Figure S17A). Even though CDK1 and CDK2 were not selected by the pruning algorithm as the most important, they are yet both present in the MoA subnetwork and the list of newly inferred targets for Dovitinib. For quizartinib, an FLT3 inhibitor, with a more limited off-target effect on FOXM1, the same procedure proposes an inhibitory effect through inhibition of CDK1 and CDK2, but also many more other potential off-targets in the subnetwork (Figure S17B). These suggest a potential trend in the model’s ability to generalize to specific classes of drugs.

**Figure 5:**
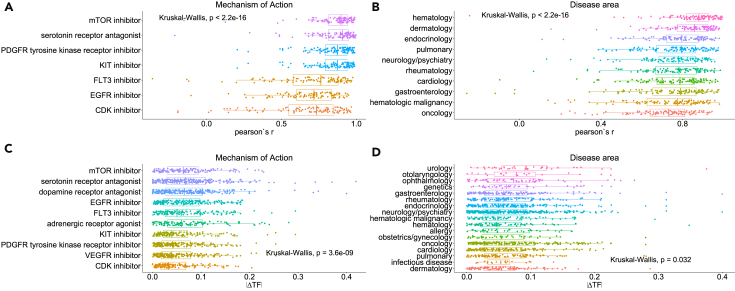
Prediction performance and off-target effects grouped by mechanisms of action and specific disease areas for the tested drugs, using the A375 cell line model (A and B) (A) Performance of every TF, across all samples and validation cell lines, grouped by the available mechanisms of action of the drugs used for evaluating the models, and (B) grouped by disease area. (C and D) (C) Drug-induced off-target effects of every TF, in every sample/drug in the A375 training cell line, grouped by the available mechanisms of action of the drugs used for training the models, including the drug module, and (D) grouped by disease areas. In all boxplots, the centerline denotes the median, the bounds of the box denote the 1st and 3rd quantiles, and the whiskers denote points not being further from the median than 1.5 x interquartile range (IQR).

Based on this we investigated trends in the models’ predictions across whole classes of drugs. We first analyzed the performance of the model and then the magnitude of the drug-induced off-target effects. The performance of TFs, across all samples and validation cell lines, indeed varies across the 7 MoAs of the test drugs (Figure 5A), which are the ones present in the top 10 most abundant (in terms of the number of available drugs) MoAs of the whole dataset, as well as across all MoAs available for the data (Figure S18A), and by disease areas (Figure 5B). This may indicate that the model is more suited for some therapeutic areas and classes of drugs, while others would need to be augmented with additional data. Similarly, we observe a significant difference across MoAs and disease areas regarding the predicted drug-induced off-target effects on TFs (Figures 5C, 5D, and S18B). This may perhaps be driven by the lack of data for some MoAs (e.g., only one drug is available for lysophospholipid receptor antagonists) or the incomplete prior knowledge of drug-target interactions. Nevertheless, these indicate that our approach could also be used to guide future experimental efforts to enrich the data and prior knowledge.

## Discussion

Drugs do not always function entirely through their proposed MoA,^6^ which may cause adverse effects from off-targets but may also sometimes be beneficial.^8^^,^^43^ Here we developed an approach for predicting the transcriptional response under drug-induced signaling, taking potential off-target effects into account. The model augments the LEMBAS framework,^29^ which simulates intracellular signaling, with a trainable module for inferring drug-induced signaling, to simultaneously predict the activity of TFs under drug stimulation and infer drug-target interactions that are not known. The model outperforms basic ML methods in predicting the TF activity. It retains most of the prior knowledge of drug-target interactions but also predicts many more putative interactions, with a good balance between sensitivity and specificity. The drug module, through its joint training with the LEMBAS framework, enables the inference of drug-target interactions that are relevant for explaining the transcriptional state of the cell, thus potentially identifying cell line-specific interactions. Perhaps, even more importantly, we make use of integrated gradients,^42^ to extract subnetworks of intracellular signaling that explain the predicted MoA of off-target effects on TF. In a case study of the drug Lestaurtinib’s off-target effects on FOXM1 activity, we demonstrated that the constructed network is biologically sensible, as we find literature support for the proposed MoA.

Understanding how the signaling effects of drugs propagate in the cell is essential for understanding how adverse effects may arise in the clinic and for designing therapy regimes that may counteract them. This is particularly important for drugs that do not function through their proposed MoA. The advent of ML and big data in biology holds promise for a more data-driven life science. However, ML models have been criticized for their lack of interpretability^54^^,^^55^ and thus many times fail to explain the underlying MoA in a biological phenomenon or were never designed to do so. Embedding prior knowledge into the structure of ML models can improve their interpretability.^29^^,^^56^^,^^57^ Specifically, in the case of our LEMBAS models, the whole architecture corresponds to feasible interactions in the intracellular signaling network. Combining this inherent structure with the inference of previously unknown drug-target interactions alongside a sensitivity approach to prune nodes and edges that do not contribute to its explanatory power, we were able to construct subnetworks that recapitulate the MoA of an off-target effect. Even further interpretability could be achieved with the integration of domain knowledge^58^ about the disease area or pathological mechanisms present in a sample, potentially allowing the utilization of underestimated drug-target interactions, whose inference is uncertain by the model.

Despite the drastic size reduction, the subnetworks explaining the MoA of off-target effects are still far too comprehensive for immediate interpretation, and, additionally, there is variation arising from the dissension between different models in the ensemble, in line with observations in the literature.^59^ While it is possible that the network indeed needs to be this large to fully recapitulate the off-target effect, this may also be the result of data limitations along with the L2 regularization used to constrain the number of inferred interactions and prevent overfitting the weights of the signaling network. Multiple drug-target interactions and paths in the network might be able to explain the observed transcriptional profile. When lacking sufficient data to train a model that can fully distinguish between all feasible solutions, this can result in the model considering multiple explanations as equally important. The former would suggest that redundancy and robustness are intrinsic to cellular circuits, which implies that a reductionistic approach in biology may lead to misleading or incomplete results. The latter would indicate that, while the biological process may be simple, we are currently too data limited to confidently simplify the network further. This problem could potentially be tackled in the future either by increasing the data used for training a model, by using large transcriptomic databases such as ARCHS4,^60^ or by using algorithms in the drug module that can indirectly infer interactions without the usage of L2 regularization.

In this study, the drug module, which infers drug-target interactions, is linear and relies on a pre-defined space of drugs and potential targets. It only uses knowledge about the chemical similarity of drugs, thereby ignoring potential structural similarities of the targets. However, the modular nature of our model allows for the future development of a drug module that can incorporate knowledge about targets’ structural similarity and is also non-linear. A previously proposed method, called DeepCE,^16^ utilizes a graph neural network^41^ to encode the chemical structure of a drug, and an attention-based ANN^61^ to combine gene-level representations, which contain gene-gene interaction information, and drug representations in a drug-gene interaction network to ultimately predict the gene expression profile of a sample. Similarly, another approach called ChemCPA^62^ also encodes the chemical structure of the drug and non-linearly scales its dose and combines it with the drug representation. On this front, our drug module could also incorporate a non-linear encoder to represent the chemical structure of drugs and combine it with targets’ representations, by building upon ideas presented in OmegaFold^63^ and AlphaFold2,^64^ in order to infer potential drug-target interactions, similar to what has been recently proposed in the ConPLex model,^65^ after training models to ultimately predict the transcriptional profile of a cell. This would expand the potential usage of our model, and especially the drug module, in a drug-target interaction screening task, for a plethora of drugs and potential targets, ultimately enabling the extensive characterization of the mechanisms of action of drug-induced off-target effects.

The present models are cell line specific and thereby do not allow an already trained model to be directly used for predictions in another cell line. In future work, this may be resolved by modeling multiple cell lines with a unified model that uses a representation of the basal state for each cell line as input, such as the sequencing profile of cell lines from the Cancer Cell Line Encyclopedia (CCLE) database.^66^ Generally, contextualizing a unified model or transferring predictions and MoA representation from one cellular model to another would be important for the utility of the model. It is important to note, however, that the use of multiple cell line-specific models can provide a consensus for the inference of a low-certainty drug-target interaction, but present in multiple models. Similarly, multiple datasets could be utilized.

Our framework introduces a way to conduct *in silico* experiments of drug perturbations while simultaneously being able to explain the MoA of a drug. As such, future use may be for designing drug combination therapies while exploring and studying their synergistic or competitive effects, identifying ways to counter drugs' off-target effects, and designing better therapeutic regimes with higher clinical efficacy.

### Limitations of the study

This work presents computer models of cellular signaling in response to drugs, based on data from cell lines. Generally, studies of model-systems can provide a more in-depth analysis compared to work on clinical samples, but, for the findings to be of therapeutic relevance, they must be translatable. A limitation of this particular study is that the models are cell line specific, and the predictions of TFs’ activities are thus not directly transferable to other cell lines. Thereby, if a target molecule is not expressed in these particular cellular models, the models may miss potential drug-target interactions, resulting in false negatives. Another limitation is that the drug module, which infers drug-target interactions, utilizes a pre-defined space of drugs and potential targets, which imposes a bound on the scope of predictions. The model makes use of chemical similarity between drugs in the inference, but it does not directly take any potential structural similarities of the targets into account.

## STAR★Methods

### Key resources table

**Table:** 

REAGENT or RESOURCE	SOURCE	IDENTIFIER
**Deposited data**		

L1000 Connectivity Map perturbational profiles from Broad Institute LINCS Center for Transcriptomics LINCS Pilot PHASE I	https://www.ncbi.nlm.nih.gov/geo/query/acc.cgi?acc=GSE92742	GSE92742
Broad’s Institute Repurposing Hub	https://repo-hub.broadinstitute.org/repurposing#download-data	Drug information: version 3/24/2020
DrugBank database, maintained by the University of Alberta and The Metabolomics Innovation Center	https://go.drugbank.com/	DrugBank (accessed on 11/3/2021)
Affymetrix microarray data from A375 melanoma cell lines treated *in vitro* with siRNAs against 45 transcription factors and signaling molecules	https://www.ncbi.nlm.nih.gov/geo/query/acc.cgi?acc=GSE31534	GSE31534

**Software and algorithms**		

R Programming language v4.1.2	R Core Team and the R Foundation for Statistical Computing	https://www.r-project.org/
Python Programming language v3.8.8	Python Software Foundation	https://www.python.org/
PyTorch framework (versions 1.10.2 & 1.12)	Linux Foundation umbrella	https://pytorch.org/
Cytoscape v3.9.1	Cytoscape Team	https://cytoscape.org/
Machine learning and downstream analysis algorithms	https://github.com/Lauffenburger-Lab/DrugsANNSignaling	https://github.com/Lauffenburger-Lab/DrugsANNSignaling

### Resource availability

#### Lead contact

Further information and requests regarding resources used and trained models’ availability should be directed to and will be fulfilled by the lead contact, Avlant Nilsson (avlant.nilsson@ki.se).

#### Materials availability

This study did not generate new unique reagents or new experimental data.

#### Data and code availability

• This paper analyzes existing, publicly available data. These accession numbers for the datasets are listed in the key resources table. Specifically, the L1000 dataset^14^ was used to train and benchmark models. The Broad’s Institute Repurposing Hub^37^ and DrugBank^13^ were used for retrieving drug-target interactions, used both in training and validation. Finally, for external evaluation, Affymetrix microarray data from A375 melanoma cell lines, treated *in vitro* with siRNAs against 45 transcription factors and signaling molecules, were retrieved from GEO (GSE31534).
• All original code has been deposited at a GitHub repository (https://github.com/Lauffenburger-Lab/DrugsANNSignaling) and is publicly available. DOIs are listed in the key resources table. In the same repository the analyzed data that were used to train our models and produce all tables and figures are also deposited.
• Any additional information required to reanalyze the data reported in this study is available from the lead contact upon request.

### Experimental model and study participant details

We not use experimental models to generate new data. The study consists computational research utilizing publicly available data.

### Method details

#### Retrieving prior knowledge networks of drug-target interactions

Drug-target interactions for training our models were retrieved from Broad’s Institute Repurposing Hub.^37^ The prior knowledge of the drug-target interactions was subset to drugs with corresponding perturbations in the L1000 dataset.^14^ Drugs were mapped with their respective targets by multiple identifiers for the drugs, namely: 1) the drugs’ SMILEs, 2) the International Chemical Identifier (InChIKey), 3) the PubChem Compound Identifier (pubchem_cid), 4) the Broad’s Institute internal identifier (pert_id), and 5) the drugs’ common names. The targets for DMSO were manually curated from DrugBank.^13^ For the evaluation of the model’s ability to retrieve drug-target interactions, we retrieved additional interactions from DrugBank,^13^ using drugs’ common names.

#### Pre-processing of *in-vitro* transcriptomics in the L1000 dataset

Transcriptomic signatures of drug perturbations were retrieved from the L1000 dataset^14^ (accessed via GEO with accession number: GSE92742). For inferring TF activity, we utilized gene expression data of 978 landmark genes, measured with the L1000 assay, and additionally, 9,196 imputed genes that were labeled as well-inferred by the L1000 study.^14^ The data were retrieved at Level 3, one of the processing steps in the pipeline of the L1000 dataset, containing normalized gene expression data. We considered only *exemplar* signatures, which, according to the L1000 definition, are the signatures with highest the transcriptional activity score (TAS) in the case of multi-signature perturbagens, i.e., technical duplicates. Briefly, the TAS metric inherent to the L1000 dataset quantifies signal strength and reproducibility, and definitions and further information are available in the CLUE platform^67^ glossary. Additionally, we keep only drugs with at least one known target in the prior knowledge signaling network (see STAR methods section for constructing the prior knowledge signaling network). After inferring TF activity and further filtering data to keep only high-quality TF activity data, we keep perturbations with at least 400 unique drugs per cell line (the number of conditions previously found to achieve high performance when training a LEMBAS signaling model^29^). After that, we keep the cell lines that have at least 200 drugs in common, so that we can construct subsequently the evaluation procedure of the model which requires common drugs tested on all these cell lines (see evaluation method section). This filtering results in 9 cancer cell lines and a drug space of 233 unique drugs. The log-scaled dose was used as input to train models ($dose_{scaled}=\log_{10}\left(dose+1\right)$). For the *in-silico* validation case study of Lestaurtinib, we utilized the level 5 *Z* score transformed data were replicates are already aggregated, and specifically for shRNA, ligand, and control (DMSO-treated and untreated cells) data we kept aggregated signatures derived from at least 3 technical replicates.

#### Pre-processing of *in-vitro* Affymetrix microarray data

For the publicly available siRNA experiments,^53^ we retrieved Affymetrix microarray data from the Gene Expression Omnibus (GEO),^52^ under the GSE31534 ascension number. The raw microarray gene expression data were normalized using the Robust Multichip Averaging (RMA) algorithm^68^ included in the *affy* R package.^69^ The normalized expression values were used to infer TF activity (see below).

#### Inference and pre-processing of transcription factor activity data

The activity of transcription factors (TFs) was inferred from transcriptomics data using the VIPER algorithm^31^ coupled with the Dorothea regulon.^30^ The VIPER algorithm calculates the enrichment of known regulons (TFs), which act as proxies of TF activity. The activity of a TF is calculated based on the expression of downstream genes known to be regulated by this specific TF, utilizing a known transcription regulatory network. The Dorothea regulon contains known regulatory interactions and thus can be used to build a regulatory network. Here we kept only high-confidence interactions (confidence levels A and B).

After inferring the TF activity of the pre-processed transcriptomic data in the L1000 dataset, we filtered TFs with high variance across technical replicates, to ensure we kept only high-quality estimations of TF activity, and then we filtered technical replicates that were not correlated enough with the other replicate signatures. To filter TFs, we first build a null distribution of TF activity variance by permuting 100 times the rows (samples) of the activity matrix, labeling this way random profiles as technical replicates, and then calculating the variance of the activity of each TF across each group of replicates. This way a null distribution of TF activity variances was built for each TF. The actual distribution of TF activity variances across replicates was compared with the null distribution, using a one-tailed Kolmogorov–Smirnov statistical test, to test whether the actual variance across replicates was less than the random variance. If the p value was greater than 0.05 the tested TF was removed and will not be utilized in downstream analysis. To filter replicates, we build a null distribution of random correlations between TF activity profiles, by randomly sampling 1000 times an equal number of signatures as the number of replicates, calculating the Pearson’s correlation between each pair and taking the mean correlation as a proxy of how similar the replicates are within a sample. We repeat this for every possible number of replicates within a sample. Then we calculate the correlation between each actual technical replicate with all others in a sample and count how many random correlations are equal to or higher than the mean correlation of the technical replicates, to calculate the probability of observing a given correlation due to change. If the p value was more than 0.05 we remove the sample and all of its replicates. Finally, we merge replicate signatures by using the median of their TF activity profiles. In case there was only one replicate, we kept the sample as it was.

#### Reconstructing a prior knowledge of signaling network

We reconstructed a prior knowledge intracellular signaling network (PKN), to constrain our ANN signaling model, from protein-protein interactions retrieved from the OmniPath database.^20^ Only human interactions from the OmniPath core set were included and further restricted to interactions originating either from the KEGG,^70^ InnateDB,^71^ or SIGNOR^21^ resources. First of all, we remove TFs and drug targets not included in the core prior knowledge network. Then we trim the PKN by removing nodes and edges from the network if for some nodes there was no path from any drug to any TF. Additionally, nodes were removed if they had only a single source and target that both were the same node. Finally, we removed TFs and drug-target interactions if a target or TF was not in the final trimmed PKN. Drugs that remained with no target in the constructed prior knowledge are removed from our data used to train and validate the model.

#### Model architecture

The model consists of two interconnected modules. First, a drug module that takes as input the concentration of a drug, in a pre-defined drug-target space, infers drug signaling. This utilizes the known drug-target interactions ($W_{DT}$) and the pre-calculated chemical similarity (denoted as $W_{sim}$ with [d x d] dimensions, where d is the number of drugs available), using the Tanimoto similarity of drugs ECFP4 fingerprints,^38^ between drugs in the drug space. Ultimately the drug signaling (S with [n x t] dimensions, where n is the number of conditions and t is the number of available targets) which is the output of the drug module is given by: $S=bn\left(X*\left(W_{sim}⊙W_{drug}\right)\right)*W_{DT}$. Specifically, the input concentration matrix (X) of available drugs is first multiplied by the element-wise product between the pre-calculated chemical similarity and a trainable weight matrix ($W_{drug}$), acting as a trainable scaler of chemical similarity, and thus controlling to which extent chemical similarity should contribute to the models’ predictions. The result of this operation is passed through a batch normalization layer^72^ with a momentum of 0.6, and, during training only, a dropout layer,^73^ with a drop-out rate of 0.1. Finally, it is multiplied with a sparse trainable weight matrix ($W_{DT}$) containing known drug-target interactions (dimensions [d x t]). The drug signaling (S) generated by the drug module, which represents the signal created by the drugs in a pre-defined drug-target space, is used as the input to the second module.

The second module is the LEMBAS framework^29^ which contains a recurrent ANN model of intracellular signaling, where the connections are based on prior knowledge of the intracellular signaling network. In LEMBAS the signaling state of each node is calculated using the signaling state of the interacting node in the previous time step, by multiplying it with a trainable connectivity matrix and adding a trainable bias, all passed through a non-linear Michaelis–Menten-like (MML) activation function, as proposed in the LEMBAS manuscript.^29^ Drug signaling (S) is first projected on the signaling nodes’ space and it is used as input in the LEMBAS network. The state vector, describing the signaling state of each node, is initialized as all 1e-3, except for TF nodes which are initialized as 0.5, and iterated for a maximum of 120 steps, after which it is assumed that a steady state has been reached. Finally, the TF activity is predicted by projecting from the signaling state of the network at the steady state.

#### Training of the model

A cell line-specific model is trained for 5000 epochs to ultimately predict the activity of 101 TFs, given the concentration of a drug, in a pre-defined drug-target space of 233 drugs and 259 potential targets. The term describing the main task of the model during training (fitLoss) is given by the Mean Squared Error (MSE) across TFs, averaged across a batch (batch size = 25) of data points used to update the weights of the model during a learning cycle. There are auxiliary terms in the training loss of the model, to constrain different parts of it, and we incorporated them from the GitHub repository (https://github.com/Lauffenburger-Lab/LEMBAS) of the LEMBAS framework,^29^ where they were originally developed. First of all, for the signaling network part of the model we want to constrain the model in biologically feasible solutions, thus the learned weights need to have the same sign as the known sign of protein-protein interaction. This is done by using a loss heavily penalizing the violation of known signs: $signConstraint=0.1*\sum_{i=1}^{V}{|w}_{i}|$, where V is the total number of violations and $w_{i}$ is a weight in the network. To prevent the fitting of parameters with extreme values, L2 regularization of the weights (NetWeightLoss) and biases (biasLoss) of the intracellular signaling network was implemented by adding the sum of squares of these vectors multiplied by 10^−6^. Additionally, to prevent weights from getting stuck at zero an additional term was added forming the final regularization term of the signaling weights as: $NetWeightLoss=10^{-6}*\sum \left(w_{i}^{2}+\frac{1}{w_{i}^{2}+0.5}\right)$. Furthermore, the trainable weights used to project from the signaling state to TF activity were also L2-regularized to avoid extreme values: $projectionLoss=10^{-6}\sum {\left(w_{pi}-1.2\right)}^{2}$. To ensure a dynamic range of signaling states for the signaling nodes in the intracellular network, we regularized the state variables so that each one of them has a uniform distribution across conditions, and this was implemented by regularizing some of the statistical properties to match the corresponding properties of a uniform distribution on the interval [0,0.99]. The regularization was implemented by calculating the deviation of the empirical properties of the distribution (mean, variance, maximum, and minimum value) across conditions from the ideal property calculated for the given interval, using the sum of squared errors. Additionally, as already described in the STAR methods section, the model was penalized with a factor of 10, when the maximum value of the signaling states was negative, and finally, all contributions were added into one term (stateLoss) and scaled in the total loss with a coefficient of 10^−5^. Finally, following the implementation proposed in the LEMBAS framework,^29^ to ensure that the model achieves convergence by reaching a steady state we aim to constrain the absolute value of the largest eigenvalue of the transition matrix, i.e., the spectral radius (ρ), to be less than 1. This is implemented with an exponential barrier function, used to constrain the spectral radius (ρ) where: $spectralRadiusLoss=\frac{1}{e^{10*\left[target\;\rho \right]}}*\left(e^{10*\rho }-1\right),\left[target\;\rho \right]=e^{\frac{\ln\left(10^{-6}\right)}{120}}$.

For the drug module, we implement two additional terms. First, we treat the drug-target interaction matrix as a small network and we regularize the weights similar to what we have done in the signaling network: $DTLoss=10^{-6}*\sum \left(w_{DT,i}^{2}+\frac{1}{w_{DT,i}^{2}+0.5}\right)$. Secondly, we implement a regularization term (DTregularization) using the trainable $W_{drug}$ matrix (described in the previous section), to control how many new interactions should be inferred and, thus how much should the model be allowed to deviate from prior knowledge by considering chemical similarity (more details in the following corresponding section). The final formula describing the total training loss, which is minimized by updating the model’s parameters using the Adam optimizer^74^ with a learning rate ranging from 10^−8^ to 2∗10^−3^ is:

$$loss=fitLoss+signConstraint+biasLoss+NetWeightLoss+DTLoss+DTregularization+10^{-3}\;*\;spectralRadiusLoss+stateLoss+projectionLoss$$

#### Evaluation of the model

To evaluate the generalization of the drug module and the LEMBAS part of the framework to unseen conditions, we implement a validation procedure where we train a whole model in the cell line with the most conditions available (VCAP), we freeze the weights of the drug module, and re-train only the signaling network part, in every one of the other 8 remaining cell lines, by using only 80% of the available drugs, while we make sure that the 20% hidden are drugs dissimilar from the ones used in training (regarding their chemical structure). If the drug module is not general enough the signaling network may change a lot and fail to generalize in dissimilar cases.

#### Regularization of the inference of drug-target interactions

To constrain the number of inferred drug-target interactions we regularize the weights of the previously described $W_{drug}$ matrix, containing trainable weights to scale the similarity between the available drugs in our data, such as that $W_{drug}$ is closer to the identity matrix (I). Thus, the regularization term used in the loss function is formed as:

$$DTregularization=\lambda_{DT}*\sum_{i=1}^{\#\;drugs}\sum_{j=1}^{\#\;drugs}{{\left(W_{drug}-I\right)}_{ij}}^{2}$$

Where $\lambda_{DT}$ is a free user-defined parameter, quantifying the strength of regularization. In this study, we performed an analysis, to study the effect of regularizing the drug-target interactions inference, with testing values from zero to infinity, where infinity, means we train a model using only the sparse trainable weight matrix ($W_{DT}$) containing known drug-target interactions. Since, the operation between $W_{drug}$ and $W_{sim}$, containing pre-calculated chemical similarity, is that of element-wise multiplication, if $W_{drug}=I$ , then $W_{sim}⊙W_{drug}=I$, meaning that the output of the drug module degenerates to: $S=X*W_{DT}$, meaning using only prior knowledge of drug-target interactions, which theoretically would be achieved with infinite regularization ($\lambda_{DT}\to \infty$).

#### The drug-target interaction inference algorithm

To infer drug-target interactions using the drug module, how much a drug affects a potential target is quantified by using integrated gradients^42^ from the Captum library^75^: $InterGrad_{i}\left(x\right)=\left(x_{i}-x_{i}^{\prime }\right)\int_{a=0}^{1}\frac{dF\left(x^{\prime }+a\left(x-x^{\prime }\right)\right)}{dx_{i}}da,x^{\prime }=baseline=0$. To identify a cut-off for identifying significantly large scores we utilize an error-based approach where we calculate the Mean Absolute Error (MAE) of the model across all TFs, after removing drug-target interactions, and thus drug input signal in LEMBAS, for increasingly higher absolute gradient score. We select as a cut-off the score that induces a 25% (or larger) increase in the model’s MAE. Drug-target interactions with a smaller score than the cut-off are considered insignificant, and thus are disregarded. Finally, we utilize the ensemble of models to derive a frequency score for each interaction appearing in multiple models and further filter the inferred drug-target interactions.

#### Node and edge importance in affecting a specific TF

To quantify the importance of a node or an edge in regulating the activity of a TF of interest we utilize a customized integrated gradient approach. First, we generate for each model the input signal from the drug module, and then we pass through the signaling module fractions of this signal’s strength, ranging from 0 to 1. We denote this input matrix $X_{in}$. The sum of the TF activity across all these artificial conditions is used as an objective function ($L_{obj}$) for which the gradients for the weights (dw) and biases (db) of the signaling module, are calculated using back-propagation. The node importance was calculated as: ${score}_{b}=\left|db\right|*\left|range\right|$, where range is the range of the node activity, for different signals’ strength, accounting this way for how sensitive a node is to changes in the signal. The range was calculated as: $range=\max\left(X_{in}\left[:,node\right]\right)-\min\left(X_{in}\left[:,node\right]\right)$. The edge importance was calculated as: ${score}_{w}=\left|dw\right|*\left|weight\right|$, where weight is the weight of an edge in the model, used in this score to account for the importance of an edge in the current trained state.

#### Identifying samples with high off-target effect

We remove the off-target signal and only the signal on the known targets is used to predict TF activities. The difference between the original predictions of the model and the ones where off-targets are masked out (Δactivity) quantifies the magnitude of the off-target effects on the TF of interest. The calculated Δactivity is derived from the mean TF activity prediction from an ensemble of 50 trained models. Samples where a TF has an activity≥0.75 or activity≤0.25, |Δactivity|≥0.2, average Pearson’s r (between training and validation) of at least 0.5, and average validation Pearson’s greater than 0.4, are considered trustworthy predictions with a large off-target effect on a specific TF. For the second step, we infer drug-target interactions for each model as previously discussed.

#### Algorithm for subsetting the network to the mechanism of action

To subset the signaling network for explaining the MoA of off-target effects edges are removed from the whole signaling network based on their importance in regulating the activity of a TF of interest. Nodes and edges are removed iteratively based on their importance (see previous section) until further removal results in the removal of all target nodes or until there is no path from the drug’s target to the TF of interest. First, we remove nodes and get rid of disconnected parts of the network, nodes that the drug cannot access through any path, and paths whose end is not the drug’s target or the TF. Then we remove edges and repeat the aforementioned network cleaning. The drug’s targets with no path to the TF are removed. Finally, we keep inferred targets that appear in at least 50% of the models, if possible, otherwise, we use a cut-off that results in at least one inferred target in that subnetwork. We do the same for edges, but if there is not a single edge that can be removed based on some frequency threshold, without maintaining the connection between some target and the TF, we use a threshold of 50% and then we start including gradually more edges to connect some target with the TF, and we keep the edges of the path with the highest sum of frequency scores (regarding the frequency in appearing in multiple models). In every part of this final trimming process, we also perform basic cleaning of the network by removing undruggable nodes, nodes that cannot affect the TF via some path, and disconnected parts of the network.

#### In-silico knockouts

To induce *in-silico* knockouts of signaling nodes, and to validate different MoAs and off-target effects, we assign a largely negative value in the input signal, that is used as input in the trained LEMBAS part of the model, to the node we wish to induce a knockout. Then this signal is propagated in the network and the model iterates for at least 120 steps, or until convergence.

#### Lethality predictions using drug-target interactions

Ten different machine Learning (ML) models (lasso, ridge regression, elastic net, random forest, XGBoost Tree, neural network, regression SVM with a linear kernel, Gaussian process, KNN, and a linear regression model from the caret^76^ library in R) are trained to predict lethality using the drug targets and cell line identity, as a one-hot encoded vector. Lethality data of drugs tested on different 8 cell lines in our study from the NCI60 drug screen^44^ were accessed via the PharmacoDB database.^77^^,^^78^ Separate ML models were trained and tested using only the prior knowledge of drug-target interactions used in the drug module of our framework and then using the inferred interaction. A Leave-One-Out-Cross-Validation (LOOCV) procedure was utilized to evaluate different models, where a drug is considered a data point, even though this might correspond to multiple samples (the same drug tested on different cell lines), and it is removed from training all samples coming from that drug. Only drugs whose targets appeared at least once in some other drug in the training data points were considered for validation.

### Quantification and statistical analysis

For the evaluation of performance in retrieving drug-target interactions (in Figure 2), metrics and the p values were calculated, through the *caret* R package,^76^ with a binomial one-tailed test comparing the proportions of accuracy and NIR.^79^ Statistical comparisons of models’ performance in terms of Pearson’s correlation were conducted using a two-sided unpaired Wilcoxon test, where asterisks are defined as: ∗∗∗∗p<=10^−4^, ∗∗∗p<=10^−3^, ∗∗p<=10^−2^, ∗p<=0.05 and ns for p > 0.05. Comparison of FOXM1 activity distributions in Figure 4 were performed with the same statistical test and asterisks notation. Non-parametric Kolmogorov-Smirnov tests are used to compare whole distributions (see the corresponding sections where they are used for details).

#### Hardware and software specifications

All models were expressed in and trained using the PyTorch framework^80^ (versions 1.10.2 & 1.12) in Python (version 3.6.13 & 3.8.8). Generally, simple simulations using one model were performed on a Dell XPS 17 laptop with an Intel i9-11900h @4.9 GHz with 8 cores (16 logic processors) and 32 GB RAM. For convenience, ensemble training of multiple models, random models training, and cross-validation was carried out on a single-threaded computer cluster (Intel Xeon CPU @ 2.60 GHz) that allowed job scheduling (using Slurm) with 16 parallel jobs. Pre-processing and statistical analysis of the results were done in the R programming language (version 4.1.2). Visualization of results was done mainly using ggplot2.^81^ More information about the versions of each library used can be found in the GitHub provided in the data and code availability section.

## Supplemental information

Document S1. Figures S1–S18 and Table S1

Data S1. Inferred drug-target interactions, alongside their ensemble frequency scores, in the A375, A549, and VCAP cell lines, related to Figure 2

Data S2. Off-target effect on TFs in A375, A549, and VCAP cell lines, together with their calculated activity and performance score, related to Figure 3
