# Supplementary material for: Inference of drug off-target effects on cellular signaling using interactome-based deep learning
Source: iScience. 2024 Mar 14;27(4):109509. doi: 10.1016/j.isci.2024.109509 (PMC11000001; doi:10.1016/j.isci.2024.109509)
Supplement: Document S1. Figures S1–S18 and Table S1 [file mmc1.pdf]

iScience, Volume *27*

Supplemental information

Inference of drug off-target effects on cellular signaling using interactome-based deep learning

Nikolaos Meimetis, Douglas A. Lauffenburger, and Avlant Nilsson

Supplementary Figures

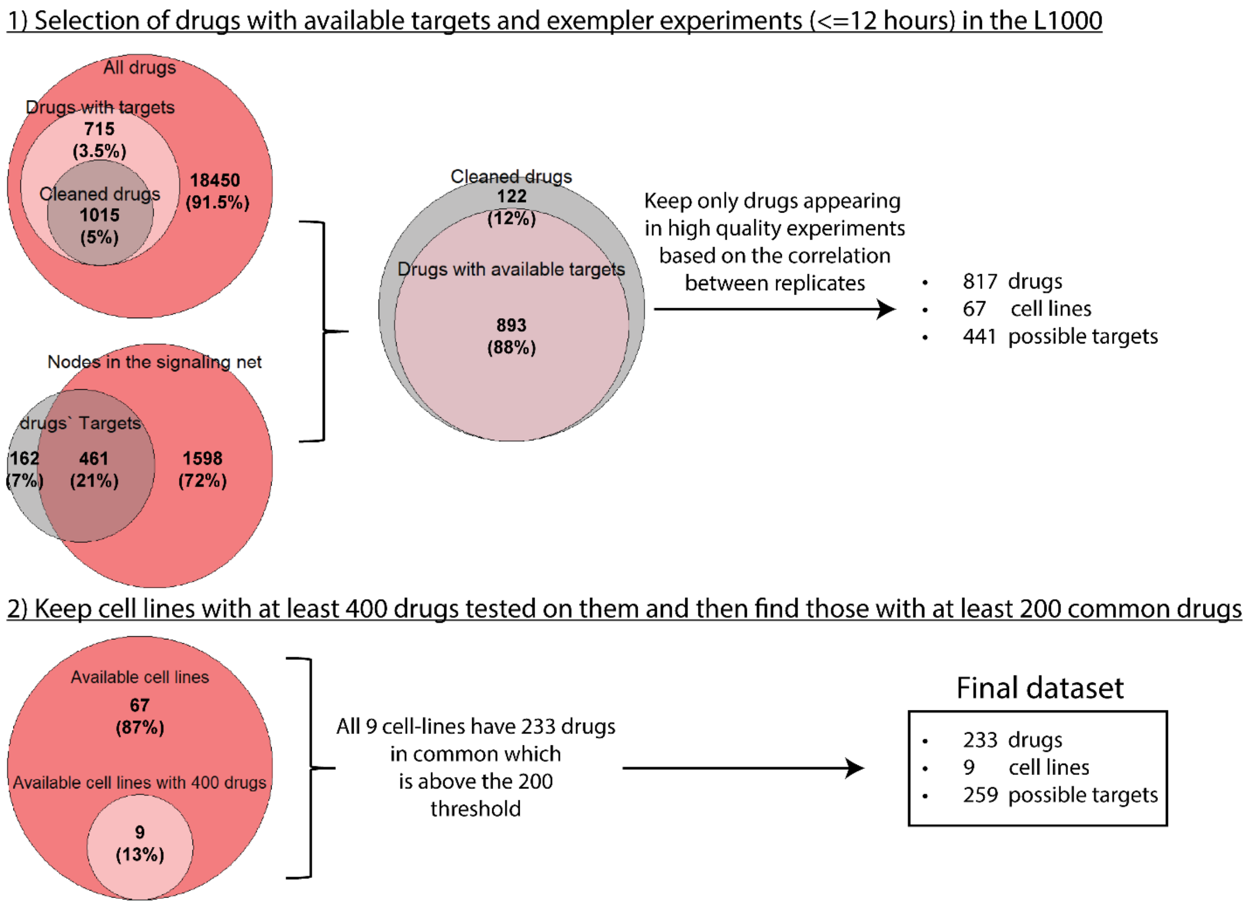

Figure S1: Data curation and selection procedure, related to STAR Methods. Schematic of the procedure used to curate the data of the L1000 dataset[1], that are used in this study for training models to predict the activity of transcription factors, while simultaneously inferring drug-target interactions. First, from the available drugs in the L1000 dataset, we keep only those with valid identifiers and available drug-target information in the Broad's Institute Repurposing Hub[2]. Then we filter down to the drugs whose targets are present in the prior knowledge signaling network used in this study, while also they are used in high-quality experiments of the L1000 dataset. Finally, we keep the data coming from cell lines with at least 400 drugs tested on each of them individually (while making sure that we keep cell lines with at least 200 common drugs), resulting in our dataset consisting of 233 drugs, tested on 9 cell lines, with 259 available targets.

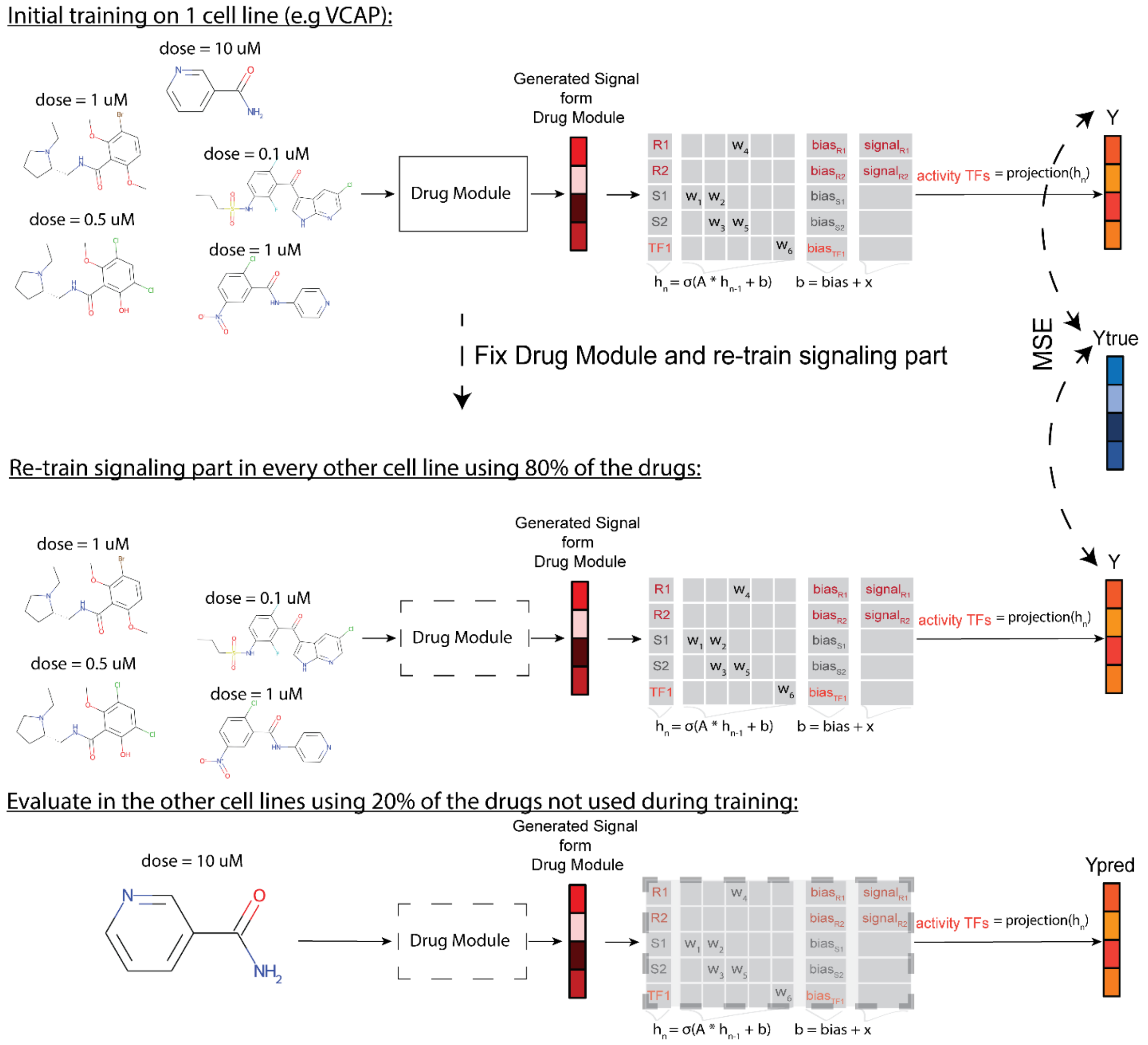

Figure S2: Training and validation procedure, related to STAR Methods. Schematic of the 3-step procedure used to evaluate the performance of the model in predicting the activities of transcription factors. Because the drug module needs to see at least once its drug but then we need to evaluate how the model performs in unseen drugs we used the illustrated procedure. First, the cell line with most samples — in this case, VCAP — is selected and a model is trained using all of its data. Secondly, the drug module's weights are fixed and only the signaling module (LEMBAS part) is re-trained on other cell lines but using only 80% of the drugs. Finally, the remaining 20% of the drugs, that were hidden were re-training the signaling module, which is used as a test set to make predictions and evaluate the performance of the model. This 20% consists of drugs that are dissimilar, in terms of chemical structure, with the drugs used in re-training the signaling module.

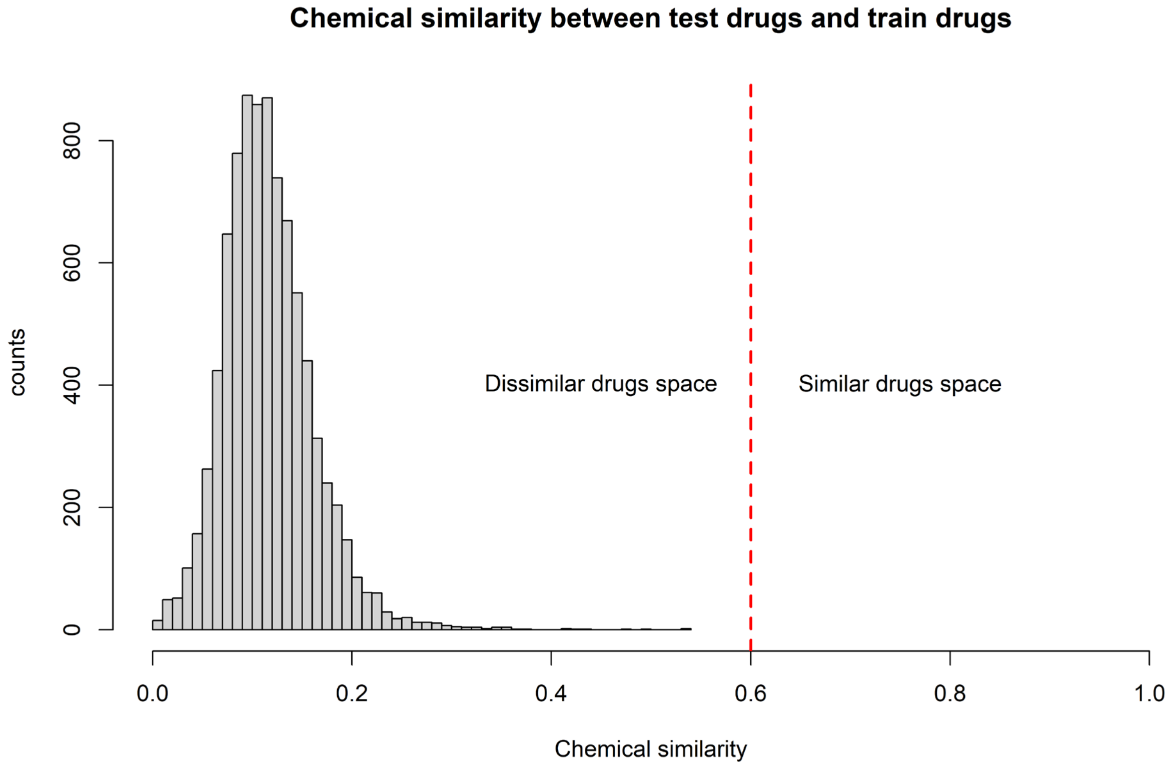

Figure S3: Chemical similarity of validation and train drugs, related to STAR Methods. Distribution of the Tanimoto similarity between ECFP4 fingerprints (quantifying the level of chemical similarity) of any test drug (20% hidden during re-training) with any other drug (80%) used in re-training the signaling part of the models.

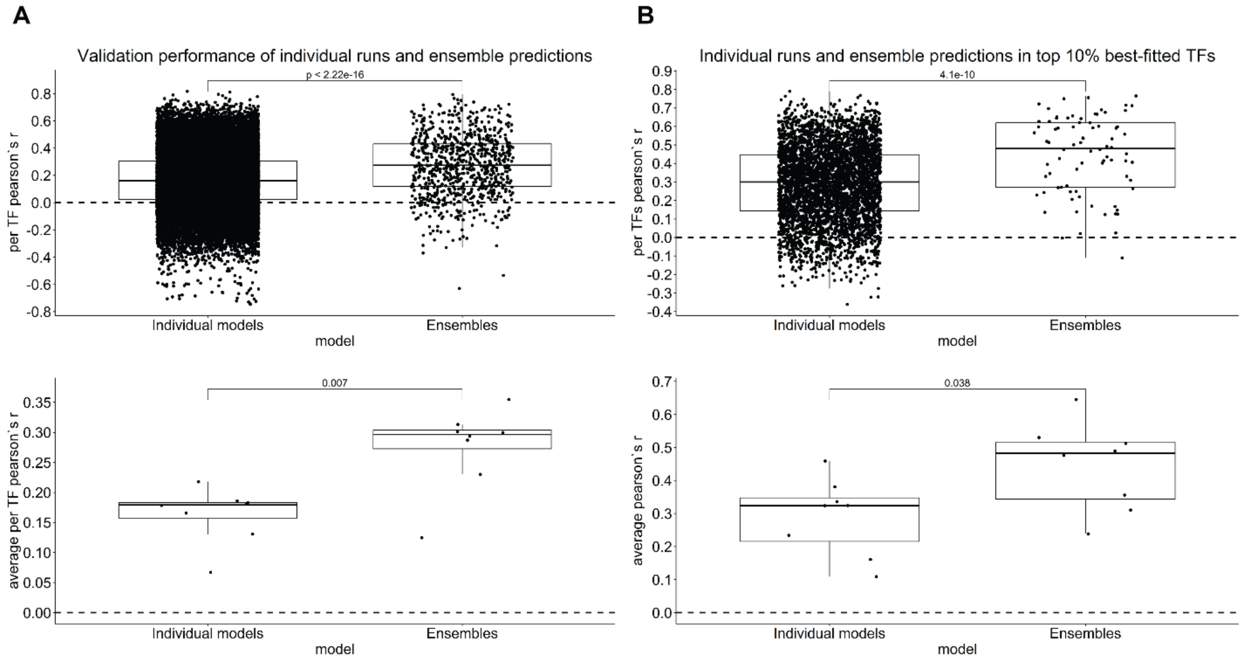

Figure S4: Performance comparison of the ensemble approach and individual models, using the VCAP cell line for initial training, related to Figure 1. A) Performance comparison of the ensemble approach and the individual models, using Pearson's r between predicted and actual TF activity **B)** Performance comparison of the ensemble approach and the individual models, by looking only at the top 10% well-fitted TFs during training. Comparisons were conducted with a two-sided unpaired Wilcoxon test.

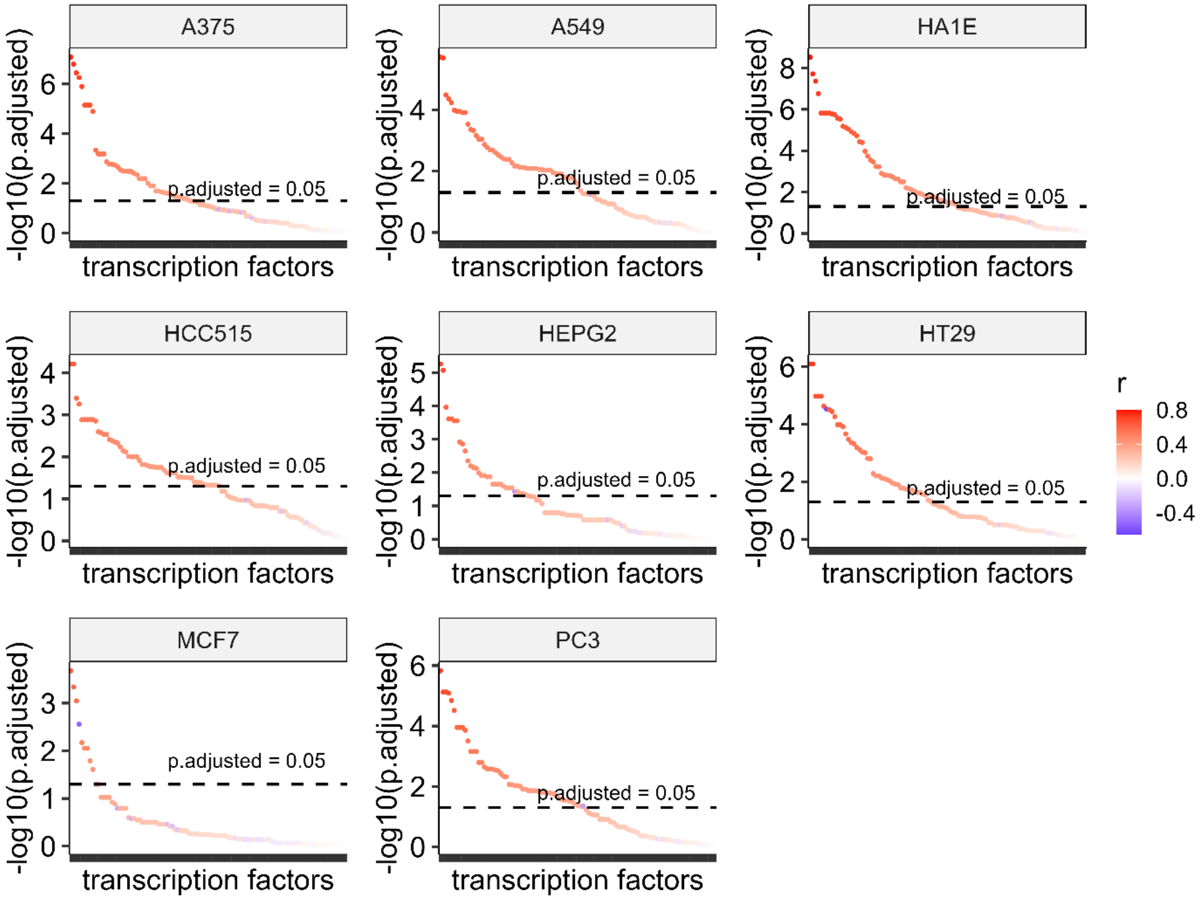

Figure S5: Adjusted p-values of the performance of individual TFs in the ensemble, related to Figure 1. The p-values from testing in each cell line if the validation Pearson's r is different from zero, for every transcription factor, were adjusted using the Benjamini-Hochberg correction.

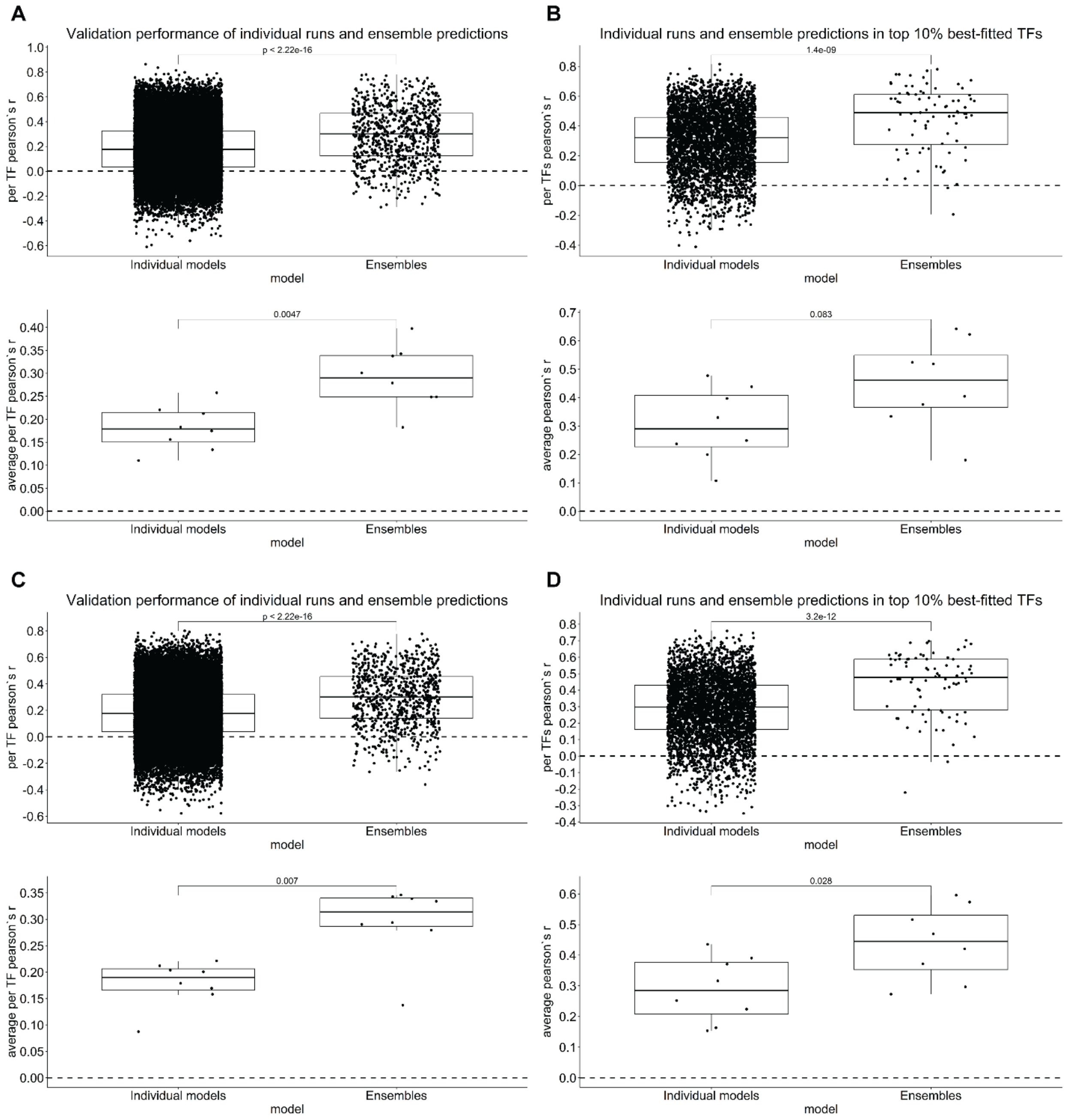

Figure S6: Performance when using the A375 and A549 as the cell line for initial training, related to Figure 1. A) Performance of ensembles and individual models when using the A375 cell line. **B)** Performance of ensembles and individual models when using the A375 cell line, by looking only at the top 10% well-fitted TFs during training. **C)** Performance of ensembles and individual models when using the A549 cell line. **D)** Performance of ensembles and individual models when using the A549 cell line, by looking only at the top 10% well-fitted TFs during training. Comparisons were conducted using a two-sided unpaired Wilcoxon test.

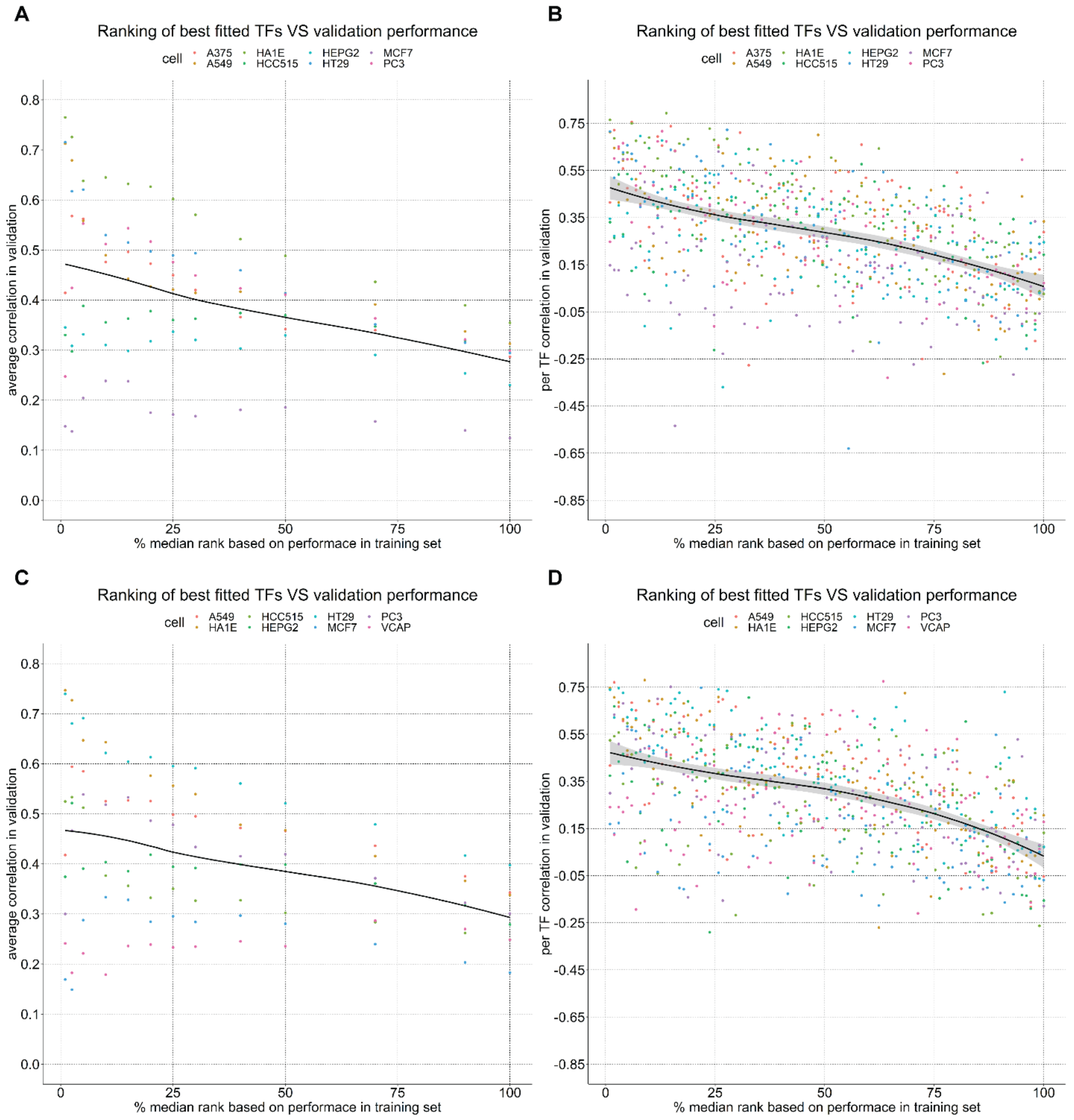

Figure S7: Validation performance when looking only at a percentage of the best-fitted TFs during training, related to Figure 1. A) Average performance across all TFs for different percentage cut-offs of training performance, when using the VCAP cell line for initial training. **B)** Performance per TF for different percentage cut-offs of training performance, when using the VCAP cell line for initial training. **C)** Average performance across all TFs for different percentage cut-offs of training performance, when using the A375 cell line for initial training. **D)** Performance per TF for different percentage cut-offs of training performance, when using the A375 cell line for initial training.

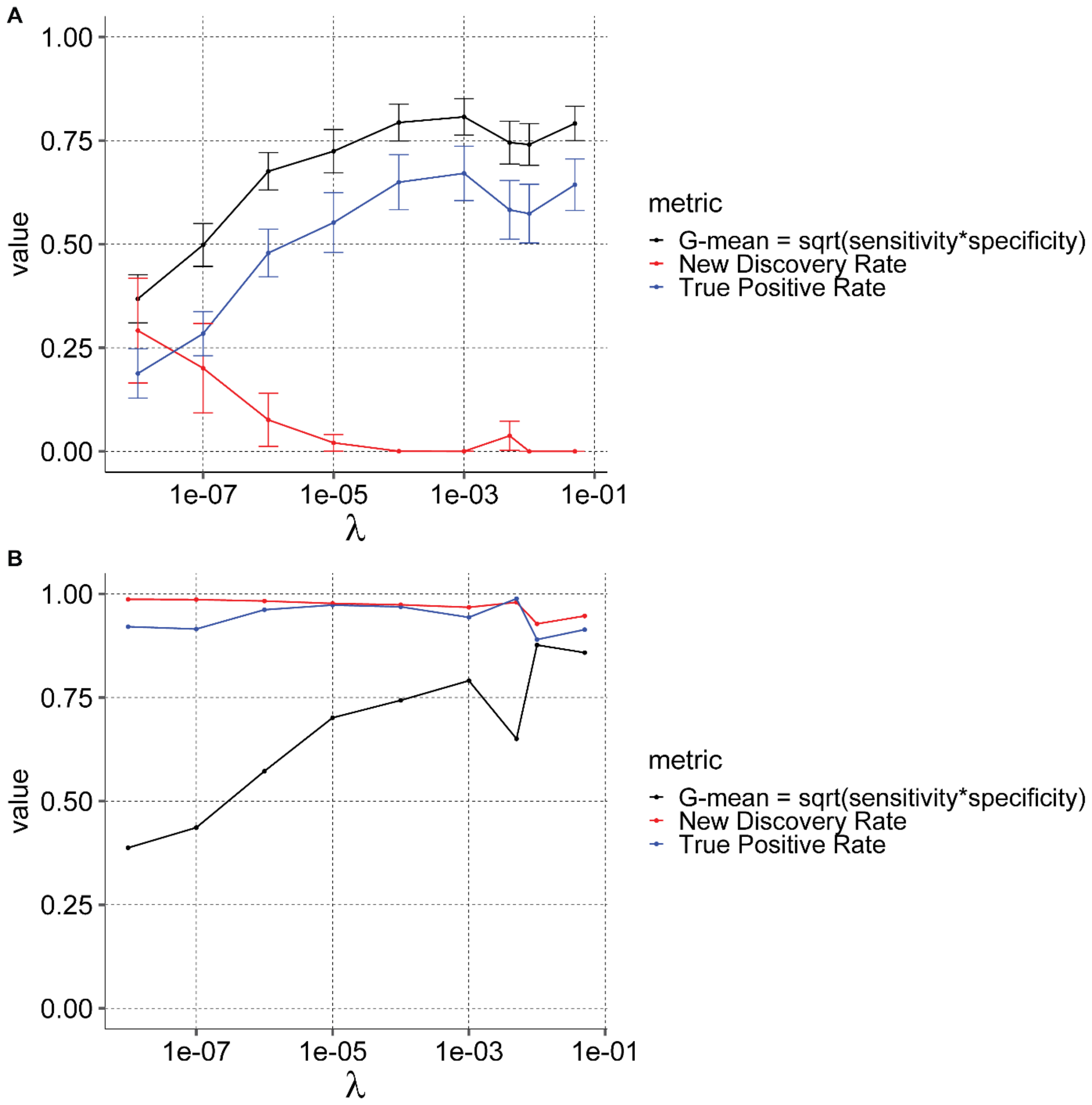

Figure S8: Inference of drug-target interactions is affected by regularization in VCAP cell line, related to Figure 1. A) The geometric mean of sensitivity and specificity (G-mean) when inferring new drug-target interactions at different levels of regularization. The error bars denote one Standard Error (SE) from the mean. The shaded area displays the 95% confidence interval around the smooth fitted line. **B)** The geometric mean of sensitivity and specificity (G-mean) and the NDR for the same gradient cut-off, when inferring new drug-target interactions at different levels of regularization, while using the error-based method discussed in the main manuscript.

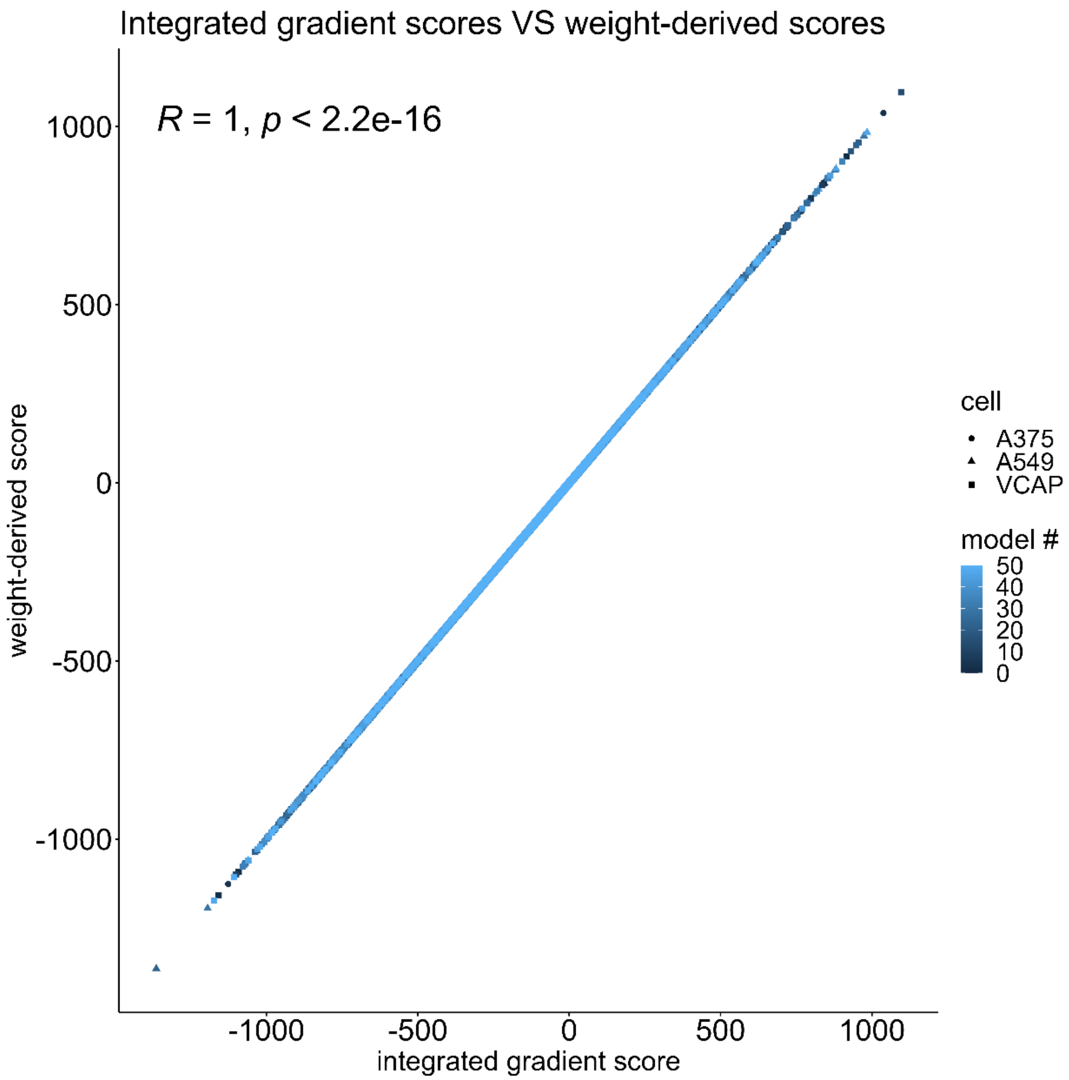

Figure S9: Relationship between the integrated gradient score[3] and the dug-target interaction weight score, related to Figure 2. The gradient score was generated from the Captum library[4], while the weight score was derived from all the linear algebra operations in the linear drug layer.

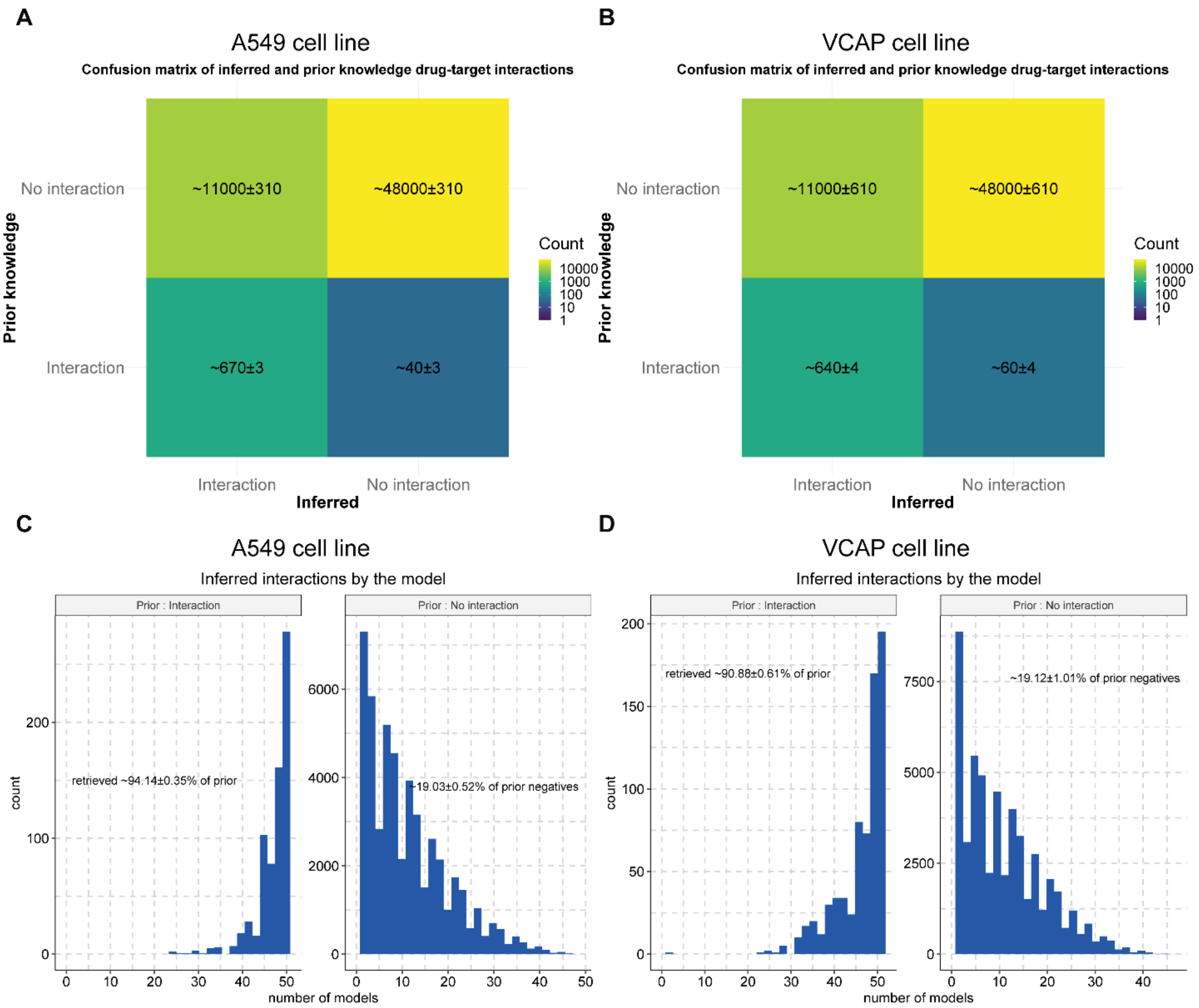

Figure S10: Inferred drug-target interactions using the drug layer, related to Figure 2. A-B) Average confusion matrix using multiple trained models for the inferred drug-target interactions. **C-D)** Percentage of prior knowledge of drug-target interactions and previously unknown interactions retrieved, and their corresponding frequency of appearance in multiple models.

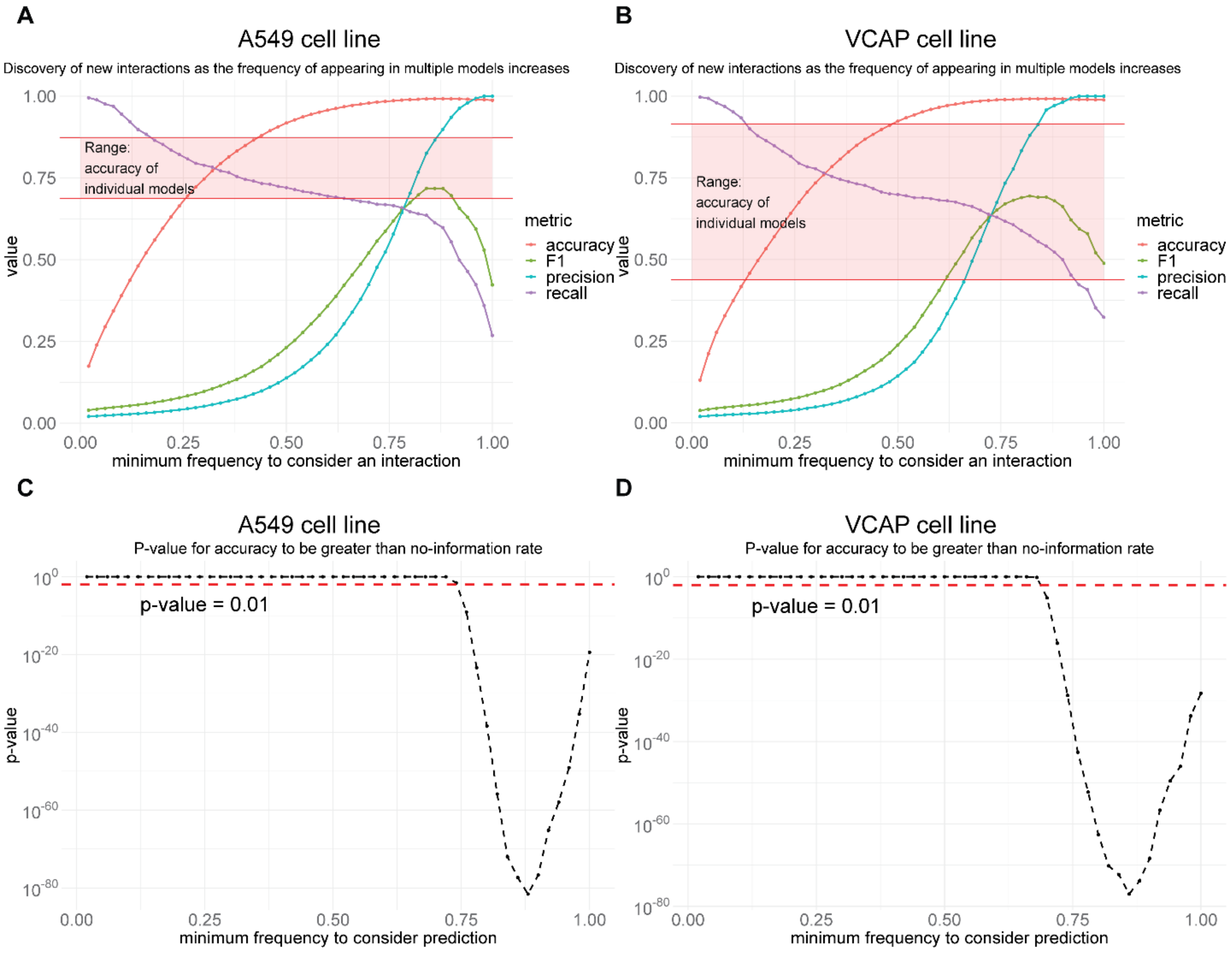

Figure S11: Inference analysis of drug-target interactions appearing in multiple models, related to Figure 2. A-B) Classification performance of our approach by considering as ground truth the interactions contained both in the Broad's Institute Repurposing Hub[2] and in DrugBank[5]. **C-D)** P-values from comparing accuracy with the accuracy obtained by assigning everything to the predominant class (No Information Rate: NIR), for multiple frequency scores

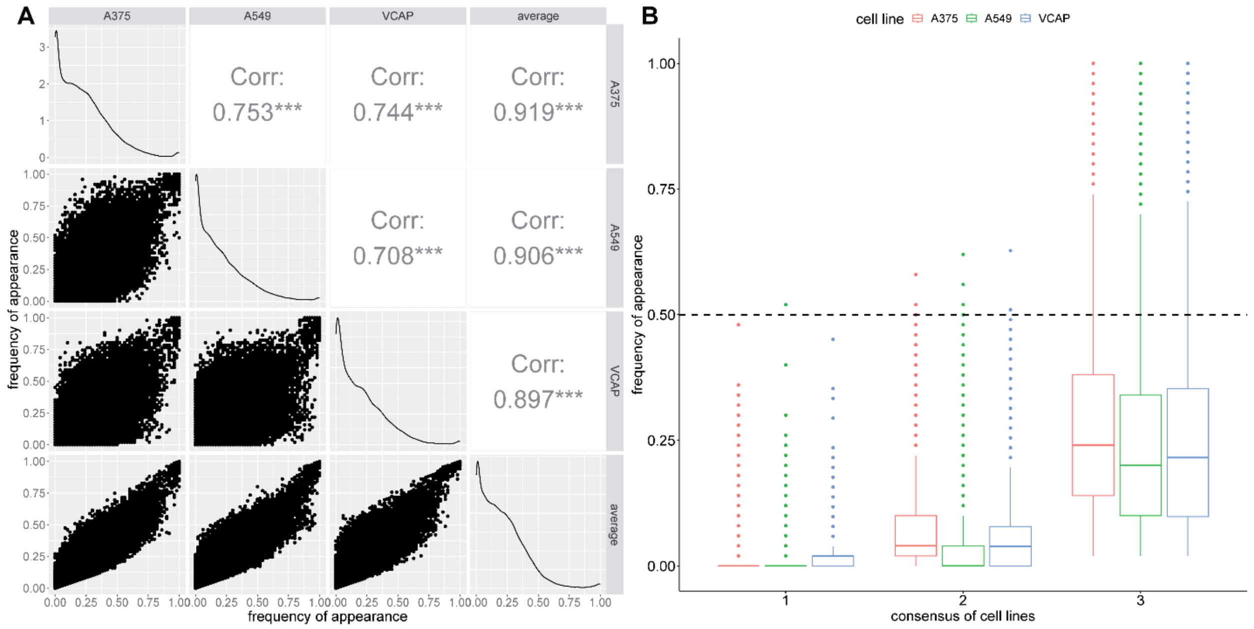

Figure S12: Frequency of appearance of inferred interactions and consensus across the three cell line-specific models, related to Figure 2. **A)** Correlation of the frequencies of appearance of inferred interactions between each cell line as well as the averaged frequency. **B)** Frequency of appearance of inferred interactions as a function of the number of cell lines that there is consensus inference.

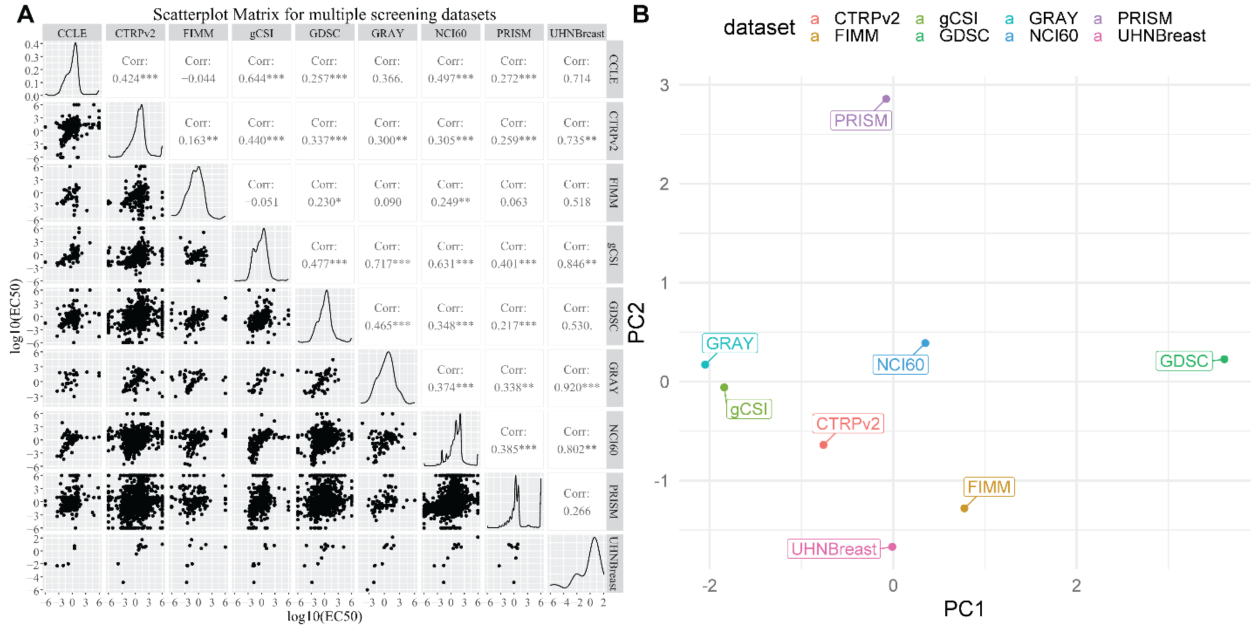

Figure S13: Comparison of the lethality values between the same perturbations between different datasets available in the PharmacoDB database[6,7], related to Figure 2. A) Correlation panel of the EC50 values between the same perturbations between different datasets. **B)** PCA plot of different datasets, with sufficiently high common number of perturbations, using EC50 values as features.

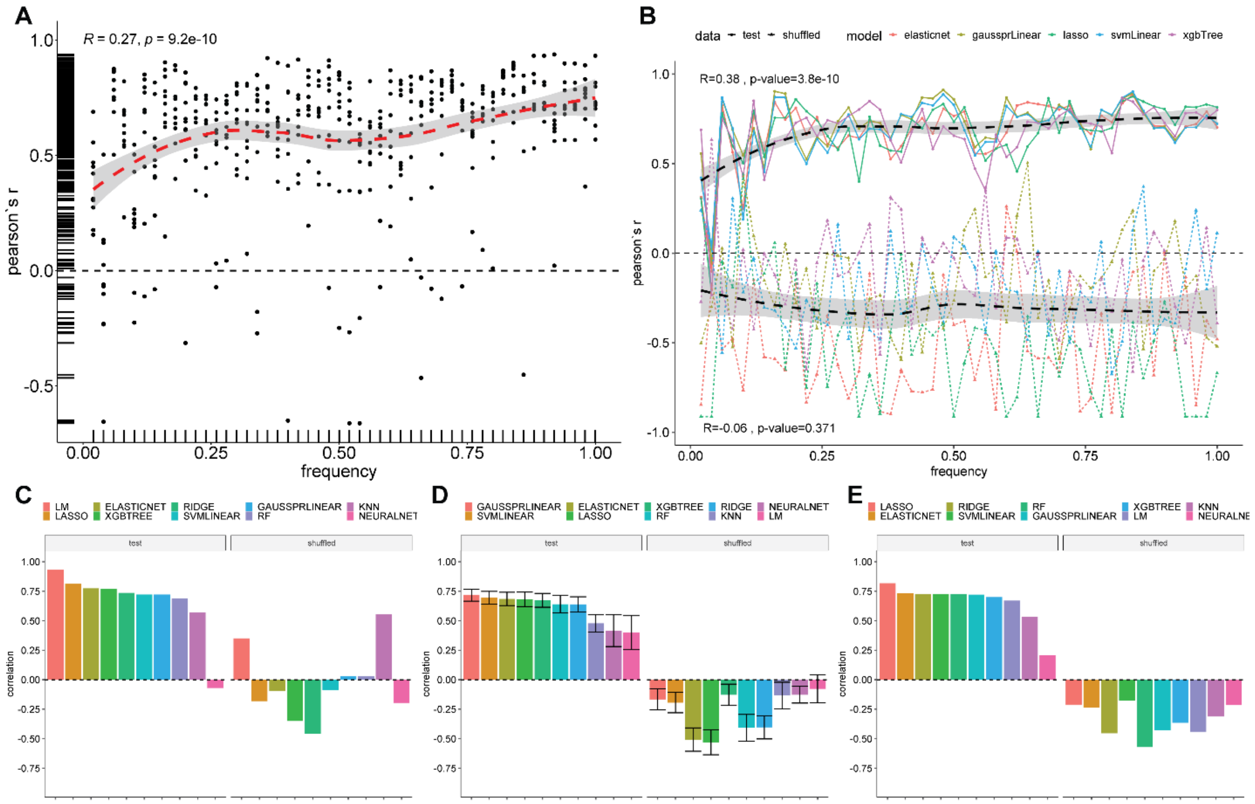

Figure S14: Performance of multiple models in predicting lethality using inferred interactions, considered by multiple models, related to Figure 2. A) Person's correlation between predicted and true lethality values, using inferred interactions across multiple thresholds of appearance and using 10 different machine learning models. **B)** Person's correlation focused on the top five models and compared simultaneously with randomized versions of these, when trained with shuffled labels. **C-E)** Barplots illustrating and comparing the performance of each of the 10 models for using as input, respectively, prior knowledge interactions (C), interactions inferred using different thresholds of frequency of appearing in ensembles of DT-LEMBAS (D), and interactions inferred using the optimal threshold (E), as identified by Figure 2D and Figures S11C-D. The error bars denote deviation of one standard error (SE) from the mean.

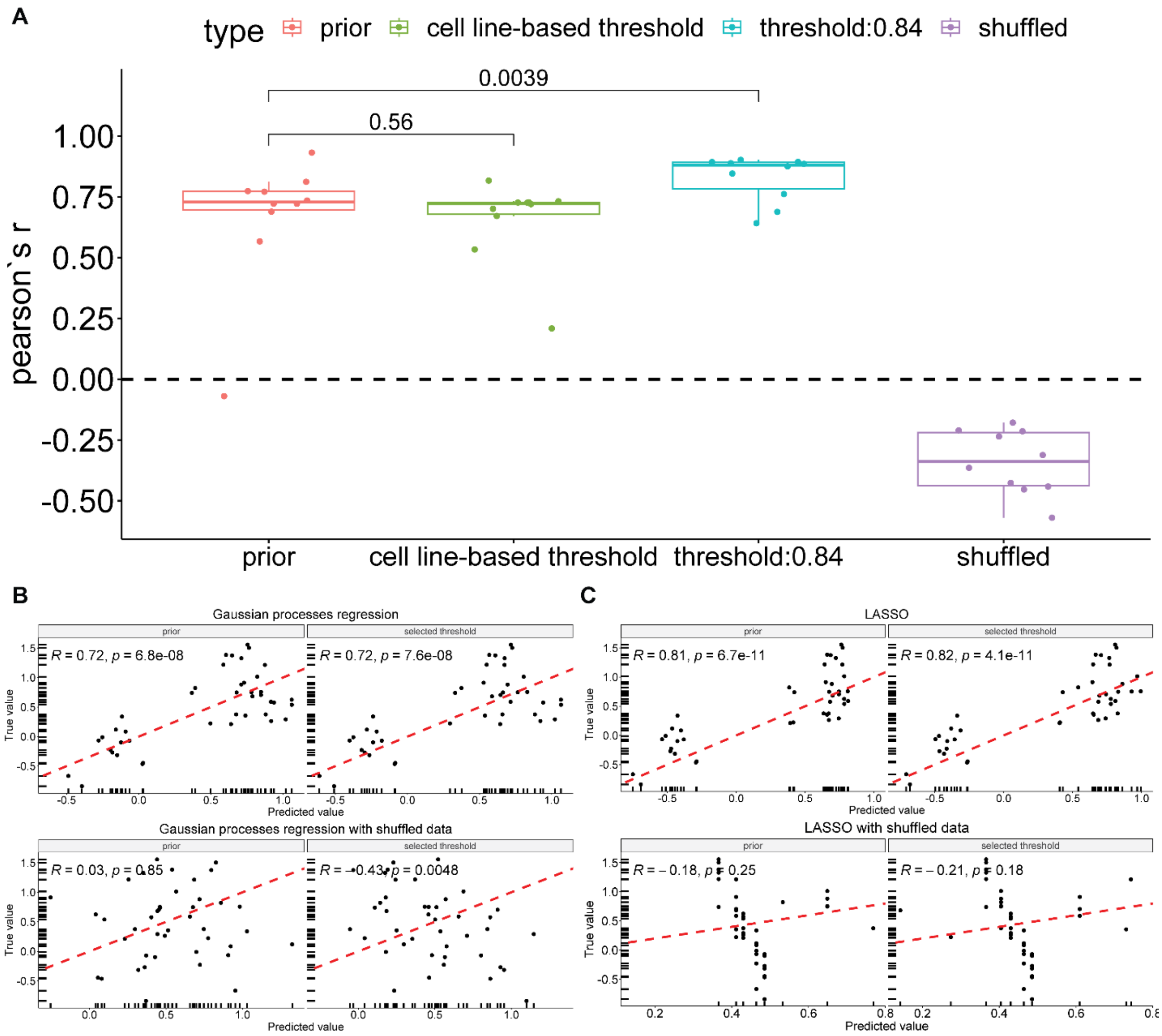

Figure S15: Comparison of input types for predicting lethality across all models, related to Figure 2. A) Performance comparison, across all ten machine learning models used for predicting lethality, when using as input types the prior knowledge interactions ("prior"), interactions inferred using the optimal threshold ("cell line-based threshold"), as identified by Figure 2D and Figures S11C-D, interactions inferred using the threshold that demonstrated the maximum performance of average ("threshold:0.84"). Additionally, we illustrate the performance of randomized models using the optimal threshold. Comparisons were conducted were compared using a two-sided unpaired Wilcoxon test. **B)** Prediction and performance, using LOOCV, of the best performing model across multiple thresholds (Gaussian Processes with a linear kernel). **C)** Prediction and performance, using LOOCV, of the best-performing model (LASSO), also appearing consistently at the top five models across all input types.

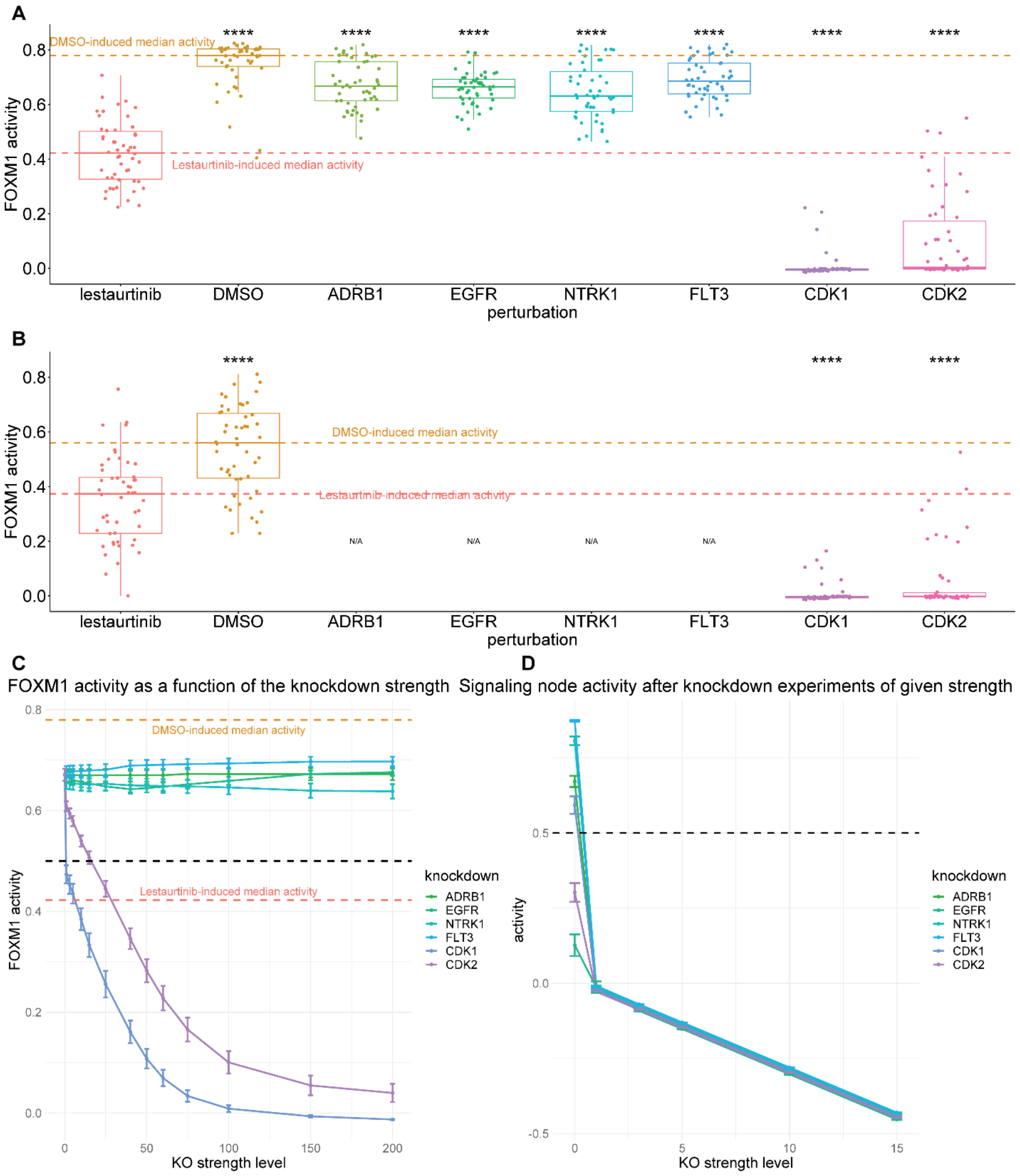

Figure S16: FOXM1 activity after *in-silico* knockdown perturbations, related to Figure 4. **A)** *In-silico* knockdown using the trained models in the A375 cell line, with a KO strength of -100. **B)** *In-silico* knockdown using the smaller inferred network explaining the MoA of the off-target effect of Lestaurtinib in the A375 cell line, with a KO strength of -100. **C)** FOXM1 activity (using the full models) for an increasing strength of KOs. **D)** Knocked-down nodes' activity for an increasing strength of KOs (using the full models). Statistical comparisons in A and B were performed relative to Lestaurtinib, with a were compared using a

two-sided unpaired Wilcoxon test, where asterisks are defined as: ****p-value<=10-4, ***p-value<=10-3, **p-value<=10-2, *p-value<=0.05, and ns for p-value>0.05.

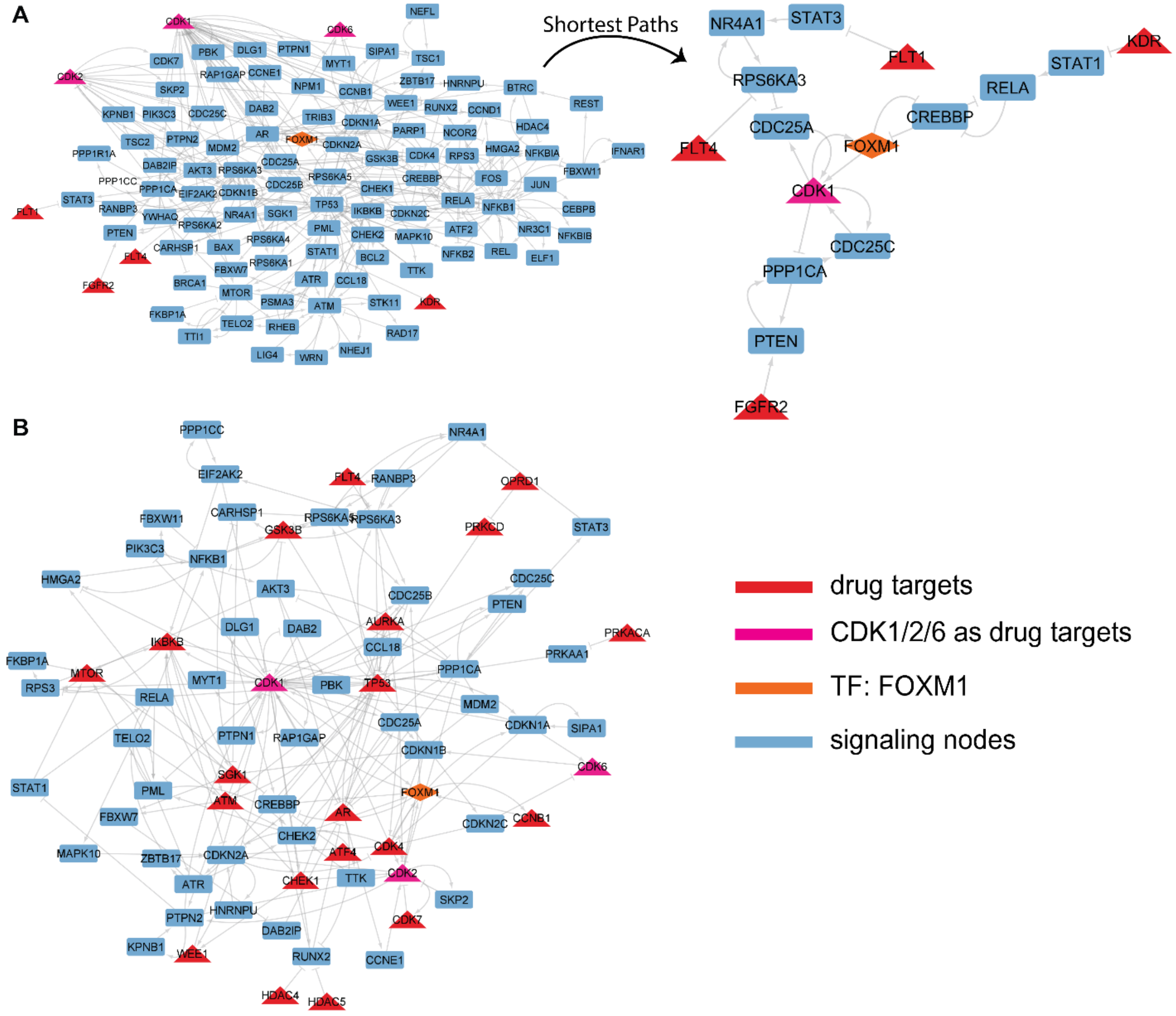

Figure S17: Proposed Mechanism of Action of the on- and off-target effects on FOXM1 of two known FLT3 inhibitors, related to Figure 3. A) The trimmed ensemble network explaining the off-target effect that leads Dovitinib to inhibit more FOXM1, via previously unknown drug-target interactions. **B)** The trimmed ensemble network explaining the small off-target effect of Quizartinib on FOXM1, via previously unknown drug-target interactions.

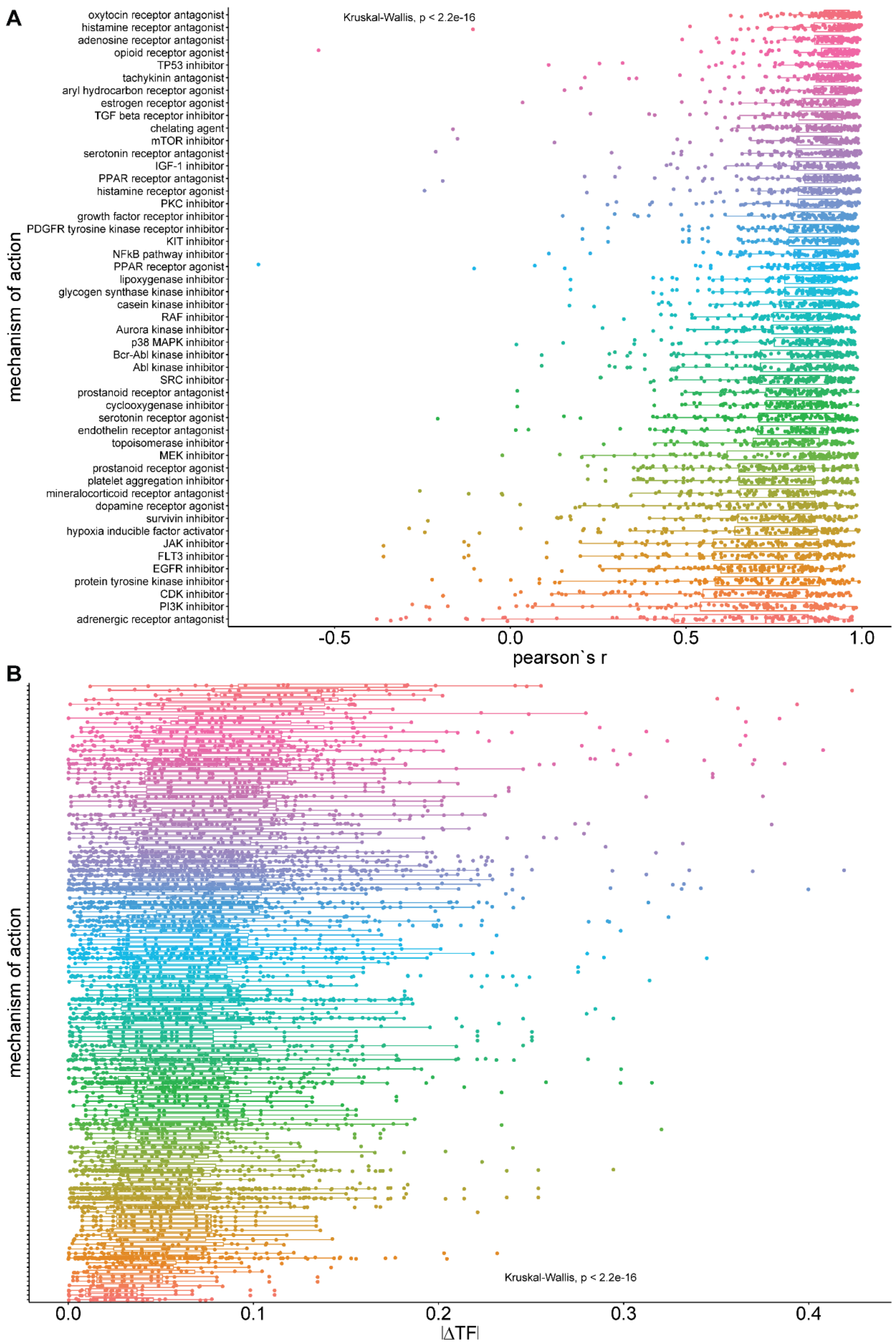

Figure S18: Model's performance and off-target effects across mechanism of actions, related to Figure 5. A) Performance of every TF, across all samples and validation cell lines, separated by the available

mechanisms of action of the drugs used for evaluating the models. **B)** Drug-induced off-target effects of every TF, in every sample/drug in the A375 training cell line, separated by the available mechanisms of action (of the drugs used for training the models, including the drug module.

Supplementary Tables

Table S1: Hyper-parameters for training a whole model, related to STAR Methods.

Parameter	Value
# of drugs	233
# of targets	259
Use precalculated ECFP4 similarity	TRUE
# of signaling nodes	2059
# of edges in the signaling net	12127
# of transcription factors	101
LEMBAS iteration steps	120
Leak (for leaky ReLU)	0.01
epochs	5000
Batch size	25
optimizer	Adam
Maximum Learning rate	$2*10^{-3}$
Minimum Learning rate	10^{-8}
Weight decay	0
Noise Level	10
L2 regularization	10^{-6}
Spectral regularization	10^{-3}

State regularization	10^{-5}
Projection L2 regularization	10^{-6}
Sign violation regularization	0.1
Target precision for spectral loss	10^{-6}
Exponential factor for spectral loss	10
inputAmplitude	3
projectionAmplitude	1.2

References

1. Subramanian, A., Narayan, R., Corsello, S.M., Peck, D.D., Natoli, T.E., Lu, X., Gould, J., Davis, J.F., Tubelli, A.A., Asiedu, J.K., et al. (2017). A Next Generation Connectivity Map: L1000 Platform and the First 1,000,000 Profiles. Cell *171*, 1437-1452.e17. 10.1016/j.cell.2017.10.049.

2. Corsello, S.M., Bittker, J.A., Liu, Z., Gould, J., McCarren, P., Hirschman, J.E., Johnston, S.E., Vrcic, A., Wong, B., Khan, M., et al. (2017). The Drug Repurposing Hub: a next-generation drug library and information resource. Nat Med *23*, 405–408. 10.1038/nm.4306.

3. Sundararajan, M., Taly, A., and Yan, Q. (2017). Axiomatic Attribution for Deep Networks. In Proceedings of the 34th International Conference on Machine Learning (PMLR), pp. 3319–3328.

4. Kokhlikyan, N., Miglani, V., Martin, M., Wang, E., Alsallakh, B., Reynolds, J., Melnikov, A., Kliushkina, N., Araya, C., Yan, S., et al. (2020). Captum: A unified and generic model interpretability library for PyTorch. Preprint at arXiv, 10.48550/arXiv.2009.07896 10.48550/arXiv.2009.07896.

5. Wishart, D.S., Knox, C., Guo, A.C., Shrivastava, S., Hassanali, M., Stothard, P., Chang, Z., and Woolsey, J. (2006). DrugBank: a comprehensive resource for in silico drug discovery and exploration. Nucleic Acids Research *34*, D668–D672. 10.1093/nar/gkj067.

6. Smirnov, P., Kofia, V., Maru, A., Freeman, M., Ho, C., El-Hachem, N., Adam, G.-A., Ba-alawi, W., Safikhani, Z., and Haibe-Kains, B. (2018). PharmacoDB: an integrative database for mining in vitro anticancer drug screening studies. Nucleic Acids Research *46*, D994–D1002. 10.1093/nar/gkx911.

7. Smirnov, P., Safikhani, Z., El-Hachem, N., Wang, D., She, A., Olsen, C., Freeman, M., Selby, H., Gendoo, D.M.A., Grossmann, P., et al. (2016). PharmacoGx: an R package for analysis of large pharmacogenomic datasets. Bioinformatics *32*, 1244–1246. 10.1093/bioinformatics/btv723.
